# Tear eicosanoids in healthy people and ocular surface disease

**DOI:** 10.1038/s41598-018-29568-3

**Published:** 2018-07-26

**Authors:** Yohannes Abere Ambaw, Cecilia Chao, Shanshan Ji, Manfred Raida, Federico Torta, Markus R. Wenk, Louis Tong

**Affiliations:** 1 Department of Biochemistry, Yong Loo Lin School of Medicine, National University of Singapore, Singapore, Singapore; 2 Singapore Lipidomics Incubator, Life Sciences Institute, National University of Singapore, Singapore, Singapore; 3 Ocular Surface Research Group, Singapore Eye Research Institute, Singapore, Singapore; 4 The School of Optometry and Vision Science, University of New South Wales, New South Wales, Australia; 5 Center For Translational Ocular Immunology, Department of Ophthalmology and Cornea Service, Tufts Medical Center, Boston, MA USA; 6 Department of Biological Sciences, National University of Singapore, Singapore, Singapore; 7 Department of Ophthalmology, Yong Loo Lin School of Medicine, National University of Singapore, Singapore, Singapore; 8 Department of Cornea and External Eye Disease, Singapore National Eye Center, Singapore, Singapore; 9 Duke-NUS Medical School, Singapore, Singapore

## Abstract

Meibomian gland (MG) dysfunction is the leading cause of evaporative dry eye and it leads to inflammation of the ocular surface. Eicosanoids may be involved in inflammation of dry eye. This study aimed to profile tear eicosanoid levels in healthy individuals and those with MG dysfunction, and to examine if these levels are associated with clinical factors and expressibility of MG. Forty participants with MG dysfunction and 30 healthy controls were recruited in this study. Clinical signs of MG dysfunction were assessed, and tear lactoferrin concentration was evaluated. Tear eicosanoids were extracted from Schirmer’s strips and analyzed using mass spectrometry. We were able to quantify 38 tear eicosanoids and levels were increased in older individuals. In participants with MG dysfunction, higher 5-HETE, LTB_4_, 18-HEPE, 12-HEPE and 14-HDoHE were associated with poorer MG expressibility. The eicosanoids PGF_2α_, 18-HEPE, 20-HDoHE and 17-HDoHE were elevated with increased corneal staining; higher 5-HETE, LTB_4_ were associated with lower tear lactoferrin levels. The receiver-operating-characteristics analysis shows higher levels of 5-HETE, LTB_4_ and 18-HEPE were able to predict poor expressibility of MGs. In conclusion, tear eicosanoid levels are age-dependent and specific eicosanoids may be indicators of clinical obstruction of MG or the severity of ocular surface damage.

## Introduction

According to the Tear Film and Ocular Surface Society Dry Eye WorkShop 2 definition, “Dry eye is a multifactorial disease of the ocular surface characterized by a loss of homeostasis of the tear film, and accompanied by ocular symptoms, in which tear film instability and hyperosmolarity, ocular surface inflammation and damage, and neurosensory abnormalities play etiological role”^1^. Dry eye syndrome affects up to 20–34% of the population worldwide, with a higher prevalence in Asians^2^, older populations and women^3^. Meibomian gland dysfunction (MGD), defined as “a chronic diffuse disease of the meibomian gland characterized by terminal duct obstruction of the meibomian glands, and qualitative and quantitative changes in the meibomian gland secretions”^4^, is a major cause of evaporative dry eye. Apart from mucin and aqueous, lipid abnormalities, including small lipids called eicosanoids, which can be measured in the tears^5^, have been reported in MGD and dry eye patients^6^.

Eicosanoids are locally acting bioactive signaling lipids derived from polyunsaturated fatty acids (PUFAs)^7^. Such metabolites are formed through the action of cyclooxygenases (COX), lipoxygenases (LOX), cytochrome P450 monooxygenases (CYP450) or free radical oxidation mechanisms^8^. Arachidonic acid (AA) (20:4n-6) is the precursor of potent mediators of the inflammatory response. COX activities generate prostaglandins (PGs)^9^, whereas LOX enzymes catalyse formation of leukotrienes (LTBs)^10^ and hydroxy-eicosatetraenoic acids (HETEs)^11^. Eicosapentaenoic (EPA) (20:5n-3) and Docosahexaenoic (DHA) (22:6n-3) acids are widely involved in the production of anti-inflammatory eicosanoids, such as hydroxy-eicosapentaenoic acids (HEPEs) and hydroxydocosahexaenoic acids (HDoHEs) respectively^12^.

Previous reports had found the presence of eicosanoids in the tear^5^. The greater the severity of meibomian gland obstruction, the more likely that lipids retained in the glands accumulate, and undergo enzymatic changes to increase levels of eicosanoids in the meibum^13^. This may then emerge as higher concentration of eicosanoid in tears. Although the association between composition of other free fatty acids in the meibum and subjective symptoms or objective signs of MGD have not been reported, increased meibum linoleic acid was found to be associated with plugging of meibomian orifices^14^. In an earlier study using ELISA, higher tear PGE_2_ and lower PGD_2_ levels were found in dry eye patients compared to the controls. Also, the PGE_2_/PGD_2_ ratio was positively associated with the severity of dry eye symptoms^5^. However, antibodies against most of the other eicosanoids were not available to assay them in the tears using ELISA, and the role of most of the eicosanoids in MGD has not been explored.

Reduction of the lacrimal protein lactoferrin in the tear is one of the indicators of aqueous deficient dry eye^15^. Since inflammation plays an etiological role in dry eye^16^, the pro-inflammatory changes on the ocular surface epithelia may include upregulation of enzymes such as COX and LOX and increased levels of downstream eicosanoids. For example, increased tear levels of 12-HETrE was associated with dry eye in humans^17^. COX-2 mRNA was significantly increased in ocular surface tissue and lacrimal glands in an experimental dry eye model in mice; and in this model PGE expression was found in periductal infiltrated cells of the lacrimal glands and conjunctival epithelium^5^. In contrast, other eicosanoids LXA_4_ and resolvin-E1, are likely beneficial as they have been shown to preserve conjunctival goblet cell viability and function^18–20^. The precursors of eicosanoids, the essential omega-3 and omega-6 fatty acids, have also been used in the treatment of dry eye^21^.

Despite these studies demonstrating important roles of eicosanoids in dry eye, no study has examined a comprehensive panel of tear eicosanoids and validated the assay technology. Furthermore, it is not known if one or more tear eicosanoids are associated with the extent of clinical occlusion of meibomian glands or with clinical measures of aqueous tear deficiency.

This study aimed to firstly develop a reproducible method of quantifying tear lipid eicosanoids, and secondly, profile the eicosanoid levels in healthy individuals in different age groups, and in participants with MGD. The possible associations between tear eicosanoid levels and clinical findings, including the expressibility of meibomian glands, were also investigated.

## Results

### Eicosanoids distribution along the wetted length and background noise of the Schirmer’s strip

**Figure 1:**
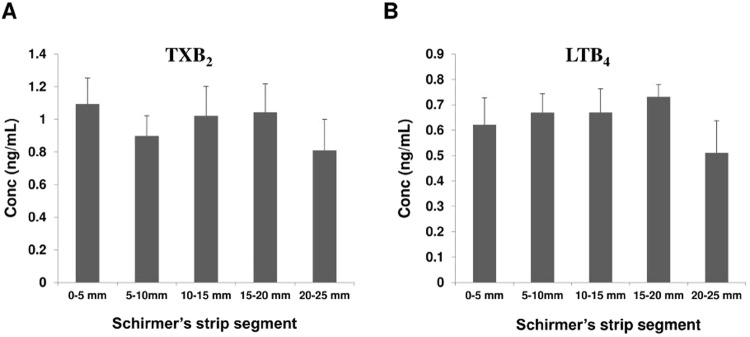
Tear concentrations of two eicosanoids, TXB_2_ (**A**) and LTB_4_ (**B**) captured within each 5 mm (0–5, 5–10, 10–15, 15–20 and 20–25 mm) segment of Schirmer’s strips. Mean values were plotted. Error bars indicate standard errors of the means. mm: millimeter.

Twenty (20) μL of mixed non-deuterated standards were applied on the edge of the Schirmer’s strip and the wetted portions of the strips were cut into 5 mm segments using micro-scissors. Lipids were then extracted from individual segments and analyzed (Fig. S3). It was found that analytes were uniformly distributed along the whole wetted length of the Schirmer’s strip. No significant differences were found between the different segments of the strip, as shown in Fig. 1. Therefore, in subsequent experiments, we have chosen to extract and analyze lipids along the entire wetted portion the strip, then normalizing to the amount of wetting +3 mm (the rounded portion of the Schirmer strip proximal to the measurement of wetting corresponds to about 3 mm of strip).

Possible lipid background signals were acquired to ensure that tears collected using Schirmer’s strips represented the real content of tears. To address the issue we considered to extract blank Schirmer’s strip and tear collected from healthy individuals (Fig S7). The blank Schirmer’s strips gave rise to relatively low background noise in the mass spectrometric analysis of the individual analytes and did not produce appreciable peak shapes in most of mass spectra (Fig S8A). This indicates that the strip materials and the plastic tubes did not contain contaminants and that our procedure was suitable for collecting tears intended for lipid analysis. However, our blank extracts also showed significant peak intensities for specific compounds, such as, 9-HODE, 13-HODE, 12,13-diHoME, 9,10-diHoME, 9-oxoODE, 13-oxoODE, 9,10,13-TriHOME, 9,12,13-TriHOME, 9,10-EpOME and 12,13-EpOME (Fig S8B). Thus, we excluded these compounds from the study.

### Tear eicosanoids in healthy participants

**Figure 2:**
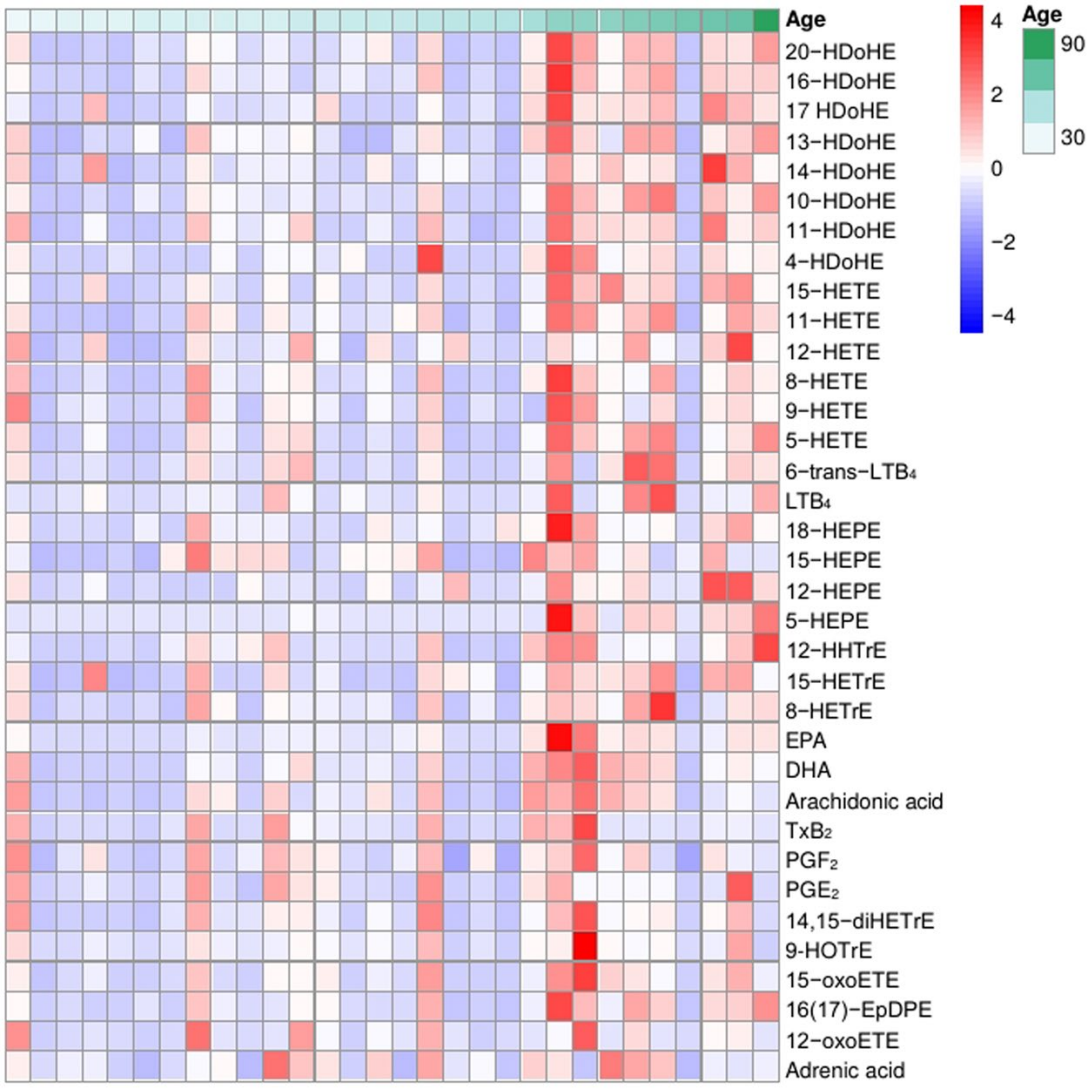
Heatmap showing the effect of age of participant and tear concentrations of eicosanoids. The color coding reflected the concentration levels of eicosanoids; red indicates high concentration, and blue indicates low concentration. Values were based on the log10 of absolute concentration (ng/mL) detected in reconstituted samples.

Thirty-eight eicosanoids were detected and successfully quantified in human tears from the healthy participants recruited (Table 1). In general, the older age group (participants above 50 years old) showed higher levels of eicosanoids (Table 1 and Fig. 2), with 25 eicosanoids out of 38 showing a significant age effect. When the participants are stratified into 3 separate age groups: 21–35 years, 36–50 years, and >50 years; twenty-four eicosanoids were significantly affected by age (Fig. S4) but 10 eicosanoids were not (Fig. S5). However, it is evident from these data (Figs S4 and S5) that the two younger age groups showed similar eicosanoid levels, which justified our previous two-level stratification. There was no difference in eicosanoid levels between genders (all p > 0.05).

**Table 1:** Tear eicosanoid levels change with age in healthy participants. n; number of subjects, age of 50 years is the overall mean age of the participants.

Eicosanoids	Younger age (<50 yr) Mean (95% CI); n = 19	Older age(>=50 yr) Mean (95% CI); n = 11	p value^ϮϮ^
**20-HDoHE**	**0.33 (0.18–0.47)**	**0.87 (0.52–1.23)**	0.001***
**16-HDoHE**	**0.2329 (0.13–0.34)**	**0.639 (0.35–0.92)**	0.001***
**17-HDoHE**	**0.56 (0.32–0.81)**	**2.16 (1.07–3.25)**	0.0001***
**13-HDoHE**	**0.05 (0.03–0.08)**	**0.13 (0.07–0.19)**	0.005*
**14-HDoHE**	**0.22 (0.14–0.31)**	**0.5 (0.27–0.72)**	0.005**
**10-HDoHE**	**0.09 (0.06–0.12)**	**0.26 (0.16–0.36)**	0.0001***
**11-HDoHE**	**0.19 (0.11–0.25)**	**0.41 (0.26–0.57)**	0.002**
**11-HETE**	**0.82 (0.56–1.10)**	**2.05 (1.28–2.82)**	0.0001***
**12-HETE**	**4.23 (2.64–5.82)**	**7.79 (4.13–11.45)**	0.03*
**8-HETE**	**0.31 (0.19–0.45)**	**0.67 (0.32–1.02)**	0.017*
**9-HETE**	**0.08 (0.03–0.13)**	**0.18 (0.07–0.29)**	0.039*
**5-HETE**	**0.33 (0.19–0.46)**	**0.96 (0.51–1.32)**	0.0001***
**15-HETE**	**1.08 (0.31–1.84)**	**2.64 (1.58–3.7)**	0.02*
**6t-LTB**_**4**_	**0.05 (0.02–0.75)**	**1.6 (0.63–0.26)**	0.002**
**LTB**_**4**_	**0.1 (0.02–0.17)**	**0.44 (0.1–0.79)**	0.004**
**18-HEPE**	**0.37 (0.02–0.05)**	**0.15 (0.03–0.27)**	0.04*
**12-HEPE**	**0.07 (0.03–0.11)**	**0.3 (0.13–0.47)**	0.001***
**5-HEPE**	**0.003 (0.0005–0.006)**	**0.114 (0.03–0.2)**	0.0001***
**5-oxo-EET**	**0.33 (0.15–0.50)**	**1.19 (0.39–1.99)**	0.02*
**15-oxo-EET**	**0.54 (0.28–0.79)**	**1.68 (0.42–2.94)**	0.008**
**16(17)-EpDPE**	**0.31 (0.18–0.43)**	**0.91 (0.51–1.31)**	0.0001***
**15-HETrE**	**0.53 (0.32–0.73)**	**1.07 (0.69–1.44)**	0.006**
**8-HETrE**	**0.07 (0.04–0.10)**	**0.18 (0.07–0.28)**	0.01*
**EPA**	**1.06 (0.66–1.45)**	**4.35 (1.40–7.30)**	0.001***
**DHA**	**1.52 (0.92–2.13)**	**3.46 (1.82–5.11)**	0.005**
TXB_2_	0.78 (0.38–1.18)	1.48 (−0.42–3.38)	0.253
PGF_2α_	0.18 (0.12–0.23)	0.21 (0.10–0.32)	0.495
PGE_2_	0.16 (0.15–0.77)	0.18 (0.02–0.5)	0.27
14,15-diHETrE	0.17 (0.1–0.24)	0.41 (0.01–0.82)	0.059
12-HHTrE	0.16 (0.09–0.23)	0.31 (0.11–0.51)	0.057
9-HOTrE	0.49 (0.35–0.64)	0.52 (0.35–0.69)	0.838
13-HOTrE	0.55 (0.38–0.72)	0.63 (0.43–0.84)	0.572
15-HEPE	0.68 (0.42–0.96)	0.88 (0.52–1.24)	0.385
12-oxoETE	0.26 (0.09–0.43)	0.44 (−0.02–0.89)	0.332
Arachidonic acid	1.45 (0.93–1.97)	2.3 (1.25–3.35)	0.088
Adrenic acid	0.019 (0.01–0.026)	0.024 (0.010–0.038)	0.453

Data presented in p values were analyzed using *t*-tests. *p < 0.05, **p < 0.01, ***p < 0.001. Concentrations of all lipids in ng/mL except Arachidonic acid (AA), Eicosapentaenoic (EPA), Docosahexaenoic (DHA) and adrenic acid. AA, EPA, DHA and Adrenic acid shown in µg/mL.

### Tear eicosanoids in patients with meibomian gland dysfunction

It is of interest to know if individual lipids were affected by clinical obstruction of meibomian glands. The tear concentrations of eicosanoids between case (reduced number of meibomian glands with liquid expression: 2 or less) and comparison groups (3 or more glands with liquid expression) are shown in Table 2.

**Table 2:** Tear eicosanoid levels between subjects with poor Meibomian gland expressibility and comparison subjects.

Eicosanoids	Case (n = 29) mean ± SD	Comparison (n = 11) mean ± SD	Fold change Case/comparison	p value
**5-HETE**	**0.69** ± **0.62**	**0.36** ± **0.62**	**1.95**	0.01**
**LTB**_**4**_	**0.14** ± **0.15**	**0.11** ± **0.26**	**1.25**	0.04*
**18-HEPE**	**0.19** ± **0.24**	**0.06** ± **0.04**	**3.13**	0.01**
**12-HEPE**	**0.62** ± **0.66**	**0.52** ± **1.06**	**1.21**	0.03*
**5-HEPE**	**0.12** ± **0.21**	**0.07** ± **0.10**	**1.88**	0.05*
**14-HDoHE**	**0.60** ± **1.18**	**0.47** ± **0.45**	**1.79**	0.05*
Arachidonic acid	3.14 ± 2.49	2.06 ± 2.62	1.52	0.08
EPA	4.54 ± 6.89	2.25 ± 2.53	2.02	0.09
9-HOTrE	2.16 ± 3.88	1.09 ± 0.76	1.98	0.14
13-HOTrE	1.42 ± 2.26	0.75 ± 0.64	1.90	0.20
17 HDoHE	0.14 ± 0.11	0.08 ± 0.05	1.83	0.09
13-HDoHE	0.10 ± 0.07	0.07 ± 0.08	1.36	0.10
11-HDoHE	0.36 ± 0.41	0.28 ± 0.45	1.32	0.10
10-HDoHE	0.17 ± 0.15	0.15 ± 0.21	1.15	0.14
20-HDoHE	0.48 ± 0.40	0.46 ± 0.59	1.06	0.36
16-HDoHE	0.33 ± 0.27	0.34 ± 0.49	9.8	0.26
4-HDoHE	0.11 ± 0.11	0.10 ± 0.17	1.03	0.21
9-HETE	0.10 ± 0.09	0.08 ± 0.12	1.26	0.11
15-HETE	1.17 ± 1.02	1.06 ± 1.32	1.11	0.22
8-HETE	0.56 ± 0.46	0.56 ± 0.89	1.01	0.13
12-HETE	1.04 ± 1.00	1.03 ± 1.74	101	0.14
11-HETE	1.35 ± 1.03	1.45 ± 2.27	9.3	0.19
6t-LTB_4_	0.09 ± 0.10	0.07 ± 0.16	1.21	0.26
12-oxoETE	0.42 ± 0.40	0.33 ± 0.52	1.26	0.16
15-oxoETE	1.20 ± 0.99	0.99 ± 1.33	1.21	0.23
PGE_2_	0.18 ± 1.53	0.13 ± 0.05	1.70	0.40
PGF_2α_	0.51 ± 0.35	0.37 ± 0.35	1.40	0.16
TxB_2_	0.43 ± 0.47	0.41 ± 0.61	1.05	0.36
16(17)-EpDPE	0.58 ± 0.37	0.42 ± 0.39	1.37	0.14
6k PGE1	0.21 ± 0.21	0.16 ± 0.21	1.32	0.21
8-HETrE	0.11 ± 0.12	0.10 ± 0.16	1.07	0.23
15-HETrE	1.06 ± 0.97	0.90 ± 1.04	1.19	0.37
12-HHTrE	0.39 ± 0.44	0.37 ± 0.55	1.05	0.26
14,15-diHETrE	0.17 ± 0.15	0.14 ± 0.21	1.17	0.12
11,12-diHETrE	0.08 ± 0.07	0.08 ± 0.11	1.09	0.16
DHA	8.57 ± 7.32	5.01 ± 4.66	1.71	0.15
Adrenic acid	0.08 ± 0.16	0.10 ± 0.25	8.0	0.15

Participants in case group had liquid secretion from 0,1, or 2 meibomian glands, definitions based on meibomian gland expressibility in the lower eyelid. Participants in comparison group had 3 or more meibomian glands showing liquid secretion (minimal or no abnormality in expressibility). Data presented in p values were analyzed using t-tests: *p < 0.05, **p < 0.01. SD; Standard Deviation. Concentrations of all lipids in ng/mL except Arachidonic acid (AA), Eicosapentaenoic (EPA), Docosahexaenoic (DHA) and adrenic acid. AA, EPA, DHA and Adrenic acid shown in µg/mL.

The results show that six of the eicosanoids were significantly elevated in participants, by 20% to 3 times higher, in participants with reduced expressibility of liquid meibum. Since these two groups had similar ages (p > 0.05) the results are not confounded by age.

Study demographic data and clinical symptoms and signs of the participants are shown in Table 3. Because of the results of Table 2, it is important to know if other clinical factors can confound the results, particularly if these clinical factors also influence meibomian gland expressibility. To further examine relationship between meibomian expressibility and Schirmer test, participant data are also stratified based on whether the Schirmer test was lower or higher than the mean of 15.45 mm. The participants who had lower MG expressibility scores, i.e., reduced number of meibomian gland with liquid secretions in the lower eyelid, as well as more MG plugs observed in the upper lids also had a lower Schirmer test (Table 3). The Schirmer test is the major clinical test used here to evaluate tear function, and a very low reading suggests aqueous tear deficiency. These results were however not adjusted by age. These findings suggest that the Schirmer test, in addition to age, may be a confounding factor for the association between meibomian gland expressibility and eicosanoids.

**Table 3:** Study demographic data and clinical symptoms and signs, presented in mean ± SD between groups based on the mean of Schirmer test of 15.45 mm.

	All (n = 40)	Low ST (n = 24)	High ST (n = 16)	P value
Sex	10 M:30 F	6 M:18 F	4 M:12 F	>0.99
Age	54.8 ± 11.5	55.9 ± 13.4	43.3 ± 8.1	0.49
Schirmer I test (mm)	**15.45** ± 14.0	**5.7 ± 3.6**	**30.1 ± 10.5**	<0.001**
Meibomian gland function and morphology assessments				
MG expressibility score	**1.1** ± 1.8	0.8 ± 1.3	1.7 ± 2.2	0.01**
Upper lid MG plugging score^Ϯ^	**2.2** ± 1.0	**2.4** ± 0.8	**1.8 ± 1.0**	0.049*
Lower lid MG plugging score	1.1 ± 0.9	1.3 ± 0.9	0.8 ± 0.8	0.09
MeiboScore (Ngo 7 scale)	1.2 ± 1.0	1.1 ± 0.8	1.4 ± 1.1	0.35
% MG dropout	34.8 ± 21.1	34.6 ± 18.6	35.2 ± 25.0	0.93
**Clinical assessments**				
SPEED	4.7 ± 5.1	4.0 ± 4.9	5.8 ± 5.3	0.28
EAPIQ	9.4 ± 9.3	8.9 ± 10.3	10.1 ± 8.0	0.70
**Lid margin telangiectasia (grade)**				
Upper lid	1.9 ± 0.9	2.0 ± 0.9	1.8 ± 0.9	0.52
Lower lid	1.5 ± 1.0	1.5 ± 0.9	1.4 ± 1.1	0.75
Corneal staining (score)	0.9 ± 1.0	1.0 ± 1.0	0.8 ± 1.1	0.54
Tear lactoferrin (mg/ml)	1.5 ± 0.4	1.4 ± 0.4	1.6 ± 0.4	0.08
TBUT (seconds)	7.34.8	7.2 ± 5.2	7.4 ± 4.4	0.92
Goblet cell density (cells/mm^2^)	87 ± 123	52 ± 64	141 ± 167	0.06
Conjunctival cell metaplasia (grade)	0.2 ± 0.3	0.1 ± 0.1	0.3 ± 0.5	0.32

MG expression and MGD plugging scores based on Arika’s grading scale^13^. TBUT; non-invasive tear film break up time, ST; Schirmer’s test, EAPIQ; Eye Allergy Patient Impact Questionnaire, SPEED; Standard Patient Evaluation of Eye Dryness. Data presented in mean ± SD was analysed using t-tests. Data presented in median (IQR) was analysed using Mann-Whitney U tests. *p < 0.05, **p < 0.01, ***p < 0.001.

**Figure 3:**
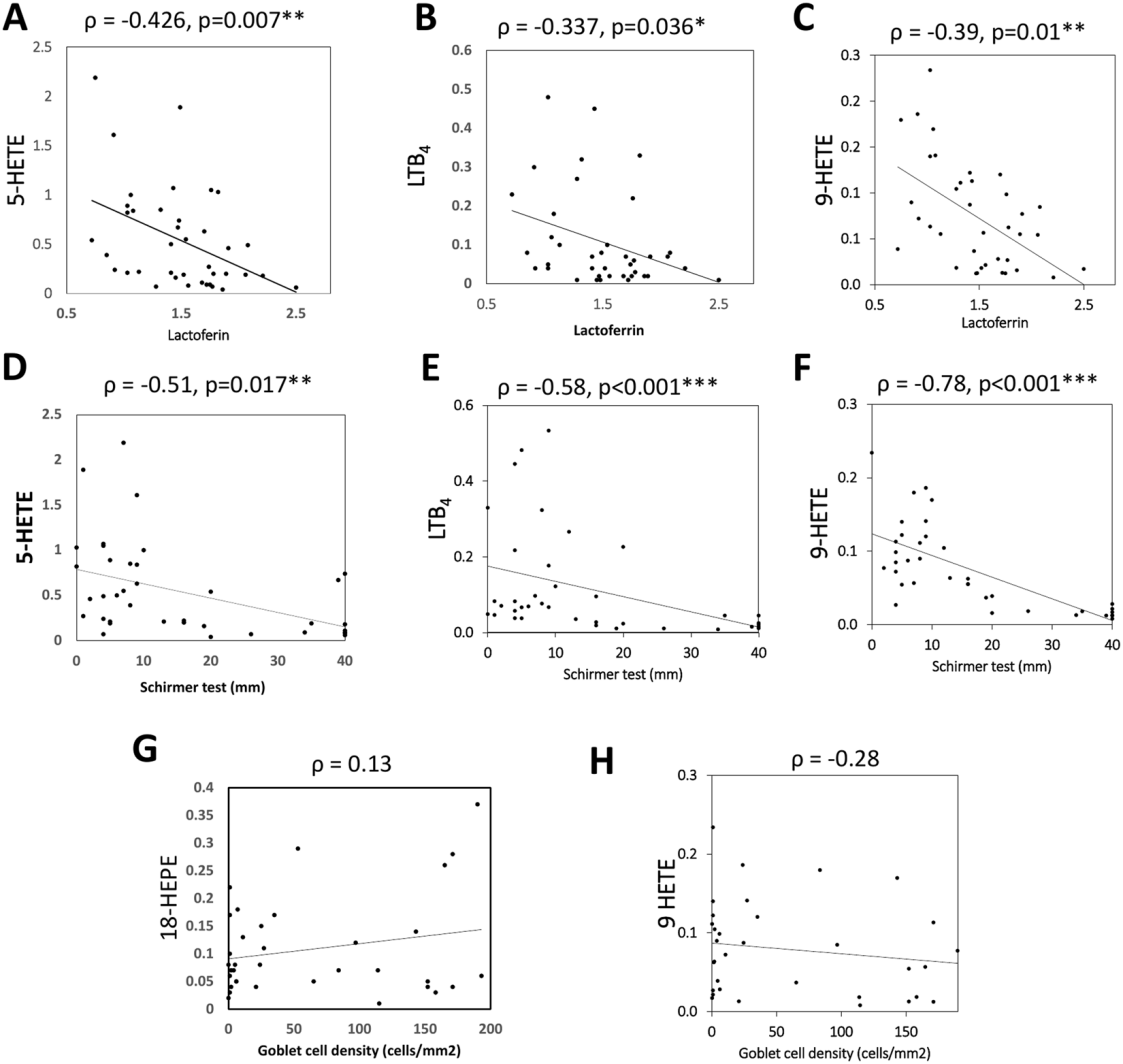
Scatter diagram showing relationship between tear eicosanoids and tear lactoferrin concentrations, Schirmer’s test results, goblet cell density scores. (**A**–**F**) tear levels of 5-HETE, 9-HETE and LTB_4_ were inversely correlated to tear lactoferrin levels or Schirmer’s test. (**G**,**H**) showing lack of correlation between two lipids and goblet cell density. ρ: spearman’s correlation coefficient rho. p values indicated significance of the correlation.

Another objective measure of lacrimal function in the tear component is the lactoferrin concentration. The scatter diagrams in Fig. 3(A–C) show that tear lactoferrin levels were inversely correlated to tear levels of 5-HETE, 9-HETE and LTB_4_ (ρ = −0.426, −0.377 and −0.39 respectively). Since these three eicosanoids were also inversely correlated to another measure of tear production, the Schirmer test (Fig. 3D–F), there is greater confidence to conclude that the three eicosanoids were elevated in the presence of poorer tear production.

Most of the eicosanoids (with only three exceptions) were correlated to the Schirmer test results (data not shown). Eicosanoid levels were not correlated to metaplasia scores or Goblet cell density scores. Two examples are shown (Fig. 3G,H).

The above results (Tables 2 and 3, Fig. 3) show that eicosanoid concentrations could be affected by multiple clinical variables in addition to age of the participants.

Consequently it is useful to perform multiple variate analyses to adjust for these variables. Based on multiple linear regression models, where each of the eicosanoid was designated as dependent variable, we identified five profiles of eicosanoids based on the number and type of clinical factors significantly influencing the eicosanoids levels (Table 4). Many eicosanoids were influenced by Schirmer test results, age and corneal staining, independently, after adjustment for each factor.

**Table 4:** Profiles of the clinical factors affecting the tear eicosanoids levels.

Profile 1	Independent variables			R^2^
	Coefficient	SE	p value	
**A. Dependent variable: PGF**_**2α**_				
Schirmer test	−0.17	0.003	<0.001***	0.57
Corneal staining	0.09	0.04	0.02*	
**B. Dependent variable: 18-HEPE**				
Schirmer test	−0.005	0.002	0.03*	0.22
Corneal staining	0.07	0.03	0.02*	
**C. Dependent variable: 20-HDoHE**				
Schirmer test	−0.02	0.004	<0.001***	0.36
Corneal staining	0.13	0.06	0.03*	
**D. Dependent variable: 17-HDoHE**				
Schirmer test	−0.003	0.001	0.001***	0.35
Corneal staining	0.03	0.01	0.01**	
**Profile 2**	**Coefficient**	**SE**	**p value**	**R** ^**2**^
**A. Dependent variable: 15-oxoETE**				
Schirmer test	−0.05	0.009	<0.001***	0.46
Age	0.03	0.01	0.02*	
**B. Dependent variable: 12-HETE**				
Schirmer test	−0.04	0.01	0.001***	0.31
Age	0.03	0.01	0.049*	
**C. Dependent variable: 9-HETE**				
Schirmer test	−0.005	0.001	<0.001***	0.45
Age	0.002	0.001	0.02*	
MG expressibility score	0.01	0.007	0.16	
**D. Dependent variable: 15-HETrE**				
Schirmer test	−0.04	0.009	<0.001***	0.39
Age	0.03	0.01	0.01**	
**E. Dependent variable: 14,15-diHETrE**				
Schirmer test	−0.008	0.002	<0.001***	0.42
Age	0.005	0.002	0.02*	
MG Plugging	−0.02	0.02	0.17	
**F. Dependent variable: 8-HETE**				
Schirmer test	−0.03	0.006	<0.001***	0.40
Age	0.02	0.007	0.03*	
MG Plugging	−0.09	0.06	0.12	
**Profile 3**	**Coefficient**	**SE**	**p value**	**R** ^**2**^
**Dependent variable: 12-HEPE**				
Non-invasive tear breakup time	−0.05	0.02	0.049*	0.28
Schirmer test	−0.03	0.008	0.001***	
Lactoferrin level	0.70	0.27	0.02*	
MG expression score	0.08	0.06	0.19	
**Profile 4**	**Coefficient**	**SE**	**p value**	**R** ^**2**^
**Dependent variable: Adrenic acid**				
Schirmer test	−0.006	0.002	0.005*	0.31
Corneal staining	0.09	0.025	0.001***	
MG Plugging score	−0.04	0.018	0.03*	
**Profile 5**	**Coefficient**	**SE**	**p value**	**R** ^**2**^
**Dependent variable: 5-HETE**				
Lactoferrin level	−0.47	0.23	0.048*	0.19
Schirmer test	−0.01	0.007	0.06	
**Dependent variable: LTB**_**4**_				
Lactoferrin level	−0.17	0.07	0.02*	0.23
Schirmer test	0.003	0.002	0.08	

Multivariate linear regression models with each lipid as the dependent variable, clinical and demographic factors were entered as independent variables. MG expressibility and plugging scores based on Arika’s grading scale^13^. SE; Standard error in the independent factor coefficient, *p < 0.05, **p < 0.01, ***p < 0.001.

Four eicosanoids were found to be associated with reduced Schirmer test results and increased corneal staining (Profile 1, Table 4). Another 6 eicosanoids were associated with reduced Schirmer test and increased age (Profile 2, Table 4).

Increased tear concentrations of 12-HEPE was associated with reduced TBUTs, reduced Schirmer tests, and interestingly, higher lactoferrin levels (Profile 3, Table 4). Two eicosanoids LTB_4_ and 5-HETE were found to be associated with reduced tear lactoferrin levels, after adjusting for Schirmer tests (Profile 5, Table 4). Higher adrenic acid levels were associated with decreased Schirmer test values and increased corneal staining, and interestingly decreased plugging score (Profile 4, Table 4).

**Figure 4:**
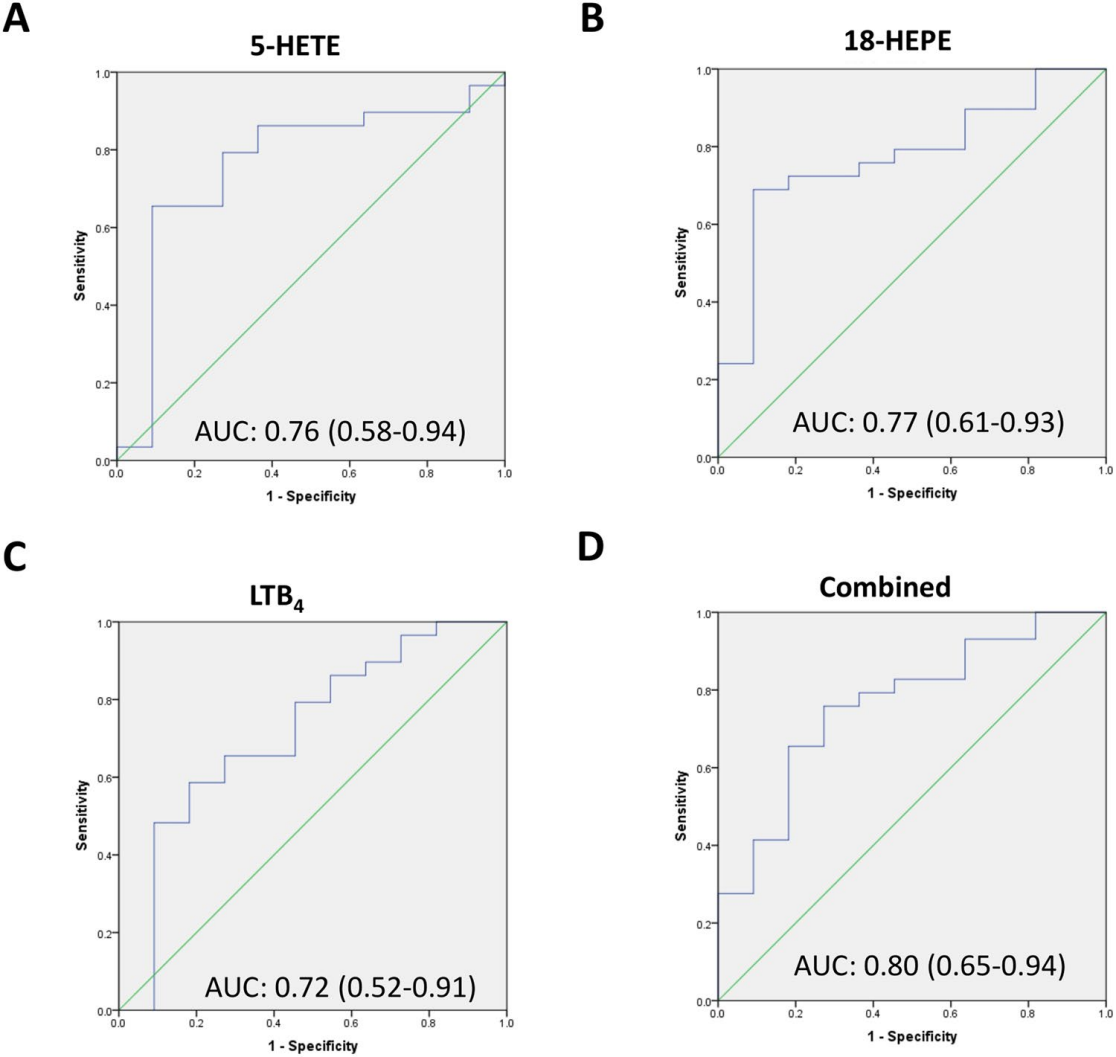
Receiver-operating-characteristic (ROC) of the three eicosanoid lipids 5-HETE, 18-HETE and LTB_4_ separately (**A**–**C**) and combined (**D**) to predict poor expressibility of liquid meibum. Diagonal line: line of no effect. The results of the ROC analysis were also shown in each panel. AUC: Area under the curve and its 95% confidence interval.

The eicosanoids in Tables 2 and 4 Profiles 3–5 are very interesting as they are not significantly associated with age, and we are interested to see if any of these lipids in isolation or in combination were able to predict the presence of meibomian gland obstruction, reflected by a reduced meibomian gland expressibility. We found that elevation of three of these eicosanoids: 5-HETE, LTB_4_ and 18-HEPE or a combination of these were able to predict reduced meibomian gland expressibility (Fig. 4).

## Discussion

We developed a novel, reproducible method of quantifying tear eicosanoids from Schirmer strips. A representative of ion chromatogram for some internal standards and endogenous species are shown in Fig. S6. In the present investigation, we found many tear eicosanoid levels to be related mainly to increased age in healthy participants, and additionally four eicosanoids increased in people with reduced Schirmer test (lower tear production) and greater corneal staining (greater ocular surface damage) (Profile 1, Table 4). These eicosanoids (PGF_2α_, 18-HEPE, 20-HDoHE and 17-HDoHE) could represent aqueous deficient dry eye. Three other eicosanoids (5-HETE, LTB_4_ and 18-HEPE) were able to predict clinical obstruction of meibomian glands and may represent evaporative dry eye or meibomian gland dysfunction.

Dennis Kwon *et al*. extracted eicosanoids from Schirmer strips with 100% methanol without SPE but only 9 eicosanoids were detectable in their samples, eg., 5-HETE only detectable in 67% in healthy and 78% in diseased eyes (cataract, glaucoma). They did not perform quantification of detected lipids. We used SPE extraction in these experiments to eliminate impurities, which significantly improves the detection of eicosanoids and quantified absolutely levels of 38 eicosanoids from human tears. Greater diversity of arachidonic acid metabolism was observed in tears of diseased eyes of children compared to normal eyes^22^. In addition, we performed additional experiments to compare Capillary and Schirmer’s tear collection techniques based on the quantity of eicosanoids recovered. Both methods essentially recovered the same species while the Schirmer’s method of tear collection showed slightly higher values in the measured amounts of eicosanoids but both methods essentially detected the same species of eicosanoids (Figs S9 and S10A). Inter-individual variation may also account for this difference, as the individuals in the 2 groups were not the same. However, the relative abundance of the analytes in the tears were very similar when comparing the two collection methods (Fig. S10B).

Another previous study^6^ collected flush tears from army veteran patients, with 93% Caucasian males, using 50 µL of saline and only 5 species of highly abundant eicosanoids were detected in the majority of samples: meibomian gland plugging score was correlated to PGE_2_ levels (R = 0.38), whereas in our study PGE_2_ levels were not correlated to plugging scores or MG expression. However, the discrepancy could be due to the different study population, with 75% of participants in our study comprised of Asian females. Moreover, in our study, participants on systemic medications that may target inflammation were excluded, whereas *Walter’s* study^6^ reported that more than 50% of the participants were on NSAIDs, and 63% were on analgesics. The use of these medications may affect levels of ocular surface inflammation and confound the results.

We found that tear eicosanoids can be associated with clinical dry eye signs or with MG expressibility. Retention of lipids in MGD may increase the action of LOX and COX-2 which leads to the increase in some proinflammatory mediators (LTB_4_, 5-HETE) and anti-inflammatory mediators (18-HEPE, 14-HDoHE). Both pro- or anti-inflammatory mediators may increase as in a cascade manner. In addition, upregulation of epithelial LOX can be found in lacrimal gland dysfunction^23^, which may result in the production of several eicosanoids. This may explain the association between reduced tear lactoferrin levels and increased lipids (18-HEPE, LTB_4_ and 5-HETE). The greater inflammatory response could be found in a more severe ocular surface epithelial damage, which may explain the association between corneal staining and inflammatory eicosanoid levels (PGF_2α_, 18-HEPE, 20-HDoHE and 17-HDoHE).

The clinical relevance is that if the topical levels of COX-2 are elevated in the epithelial cells, administration of non-steroidal anti-inflammatory drugs (NSAID) eyedrops^24^ may reduce these proinflammatory PGs and achieve relief in dry eye. Selective inhibition of COX-2 or LOX is possible using currently available drugs^25^, which suggests that tear levels of patients may be used to select candidates for treatment.

The strengths of this study are that absolute concentrations of 38 eicosanoids were successfully quantified in this study and correlations to specific clinical parameters relevant to dry eye and meibomian gland dysfunction reported. We did not examine the levels of other meibum lipids such as O-Acyl-ω-Hydroxy fatty acids and lysophospholipids, which have been found to be associated with dry eye and MGD as previously reported^26^.

It would be useful to study the changes in the pro-inflammatory and pro-resolution eicosanoids in a longitudinal fashion after treatment of MGD or after anti-inflammatory agents. Panels of eicosanoids may serve as biomarkers to identify patients for anti-inflammatory treatment or treatment to relieve MG occlusion. Since dry eye is often caused by systemic inflammation, eicosanoid levels in tears could also be used to monitor systemic diseases in personalized medicine^27^.

In conclusion, we reported tear levels of 38 eicosanoids in healthy individuals and participants with meibomian gland dysfunction. We found age, Schirmer test, corneal staining, expressibility of meibomian glands and tear lactoferrin levels to be associated with various eicosanoids. Different eicosanoids may reflect underlying disease mechanisms, eg., aqueous deficient versus evaporative dry eye.

## Materials and Methods

### Reagents

All twenty four deuterated internal standards were purchased from Cayman Chemical (Ann Arbor, MI, USA). Optima LC–MS grade acetonitrile (ACN), methanol (MeOH), and Isopropanol (IPA) were obtained from Fisher Scientific (Hampton, USA). Acetic acid was obtained from Baker analyzed A.C.S. Reagent (PA, USA).

### Study design and participants

There were two experiments in this study. Thirty healthy non-dry eye participants, aged 21 or above were recruited for the first experiment to examine the effect of age on tear eicosanoid concentrations. For the second experiment, forty participants who aged 30 years or older were recruited. Study visits were conducted at least 2 hours after awakening in order to minimize the potential diurnal variation^28–30^. All subjects were free from anti-inflammatory eyedrops (e.g. cyclosporine) or anti-inflammatory medication at least 1 month prior to the study visit. Exclusion criteria of these experiments included active eye infection, systemic disease likely to affect the ocular surface (e.g. diabetes, thyroid disease), pregnancy, or breast-feeding during the study visit.

These experiments adhered to the tenets of the *Declaration of Helsinki for human research*, and had ethics approval from the centralised SingHealth Institutional Review Board. Written informed consent was obtained prior to participation.

### Determination of the effect of age in healthy volunteers

Subjects were divided into two age bin (21–50 years and >51 years). Tears were collected from both eyes without anaesthesia using the Schirmer’s strips (Bausch and Lomb® Sno Strips, New York, NY) and the strips were stored at −80 °C in glass vials until batch analysis of tear eicosanoid concentrations (see lipid extraction, instrumentation and analysis sections). The collection time was limited to a maximum of 5 min with collection volumes of less than 10 μL tear liquid per sample.

### Determination of the effect of clinical factors in MGD

The Standard Patient Evaluation of Eye Dryness (SPEED)^31^ and the occurrence of eye allergy symptoms section in the Eye Allergy Patient Impact Questionnaire (EAPIQ)^32^ were used to assess dry eye and ocular allergy symptoms respectively. Higher scores in the questionnaires indicate greater intensity/frequency of the symptoms suffered.

In each participant, the worse eye of the two was selected for this study, based on the MG expression and plugging score as previously described^13^. MG expression was examined using a standard force MG evaluator (TearScience, Morrisville, NC) at the central sector of the lower eyelids on both eyes visualized under a slit lamp biomicroscope^33^, and the number of glands with expression of liquid meibum counted. MG obstruction on the upper and lower eyelids was separately documented using a scale based on the number of visibly blocked gland orifices (Grade 0–3, where 0 = no obstruction in any gland, 3 = totally blocked orifices in most glands)^13^. The lid margin telangiectasia score (Grade 0–3) was used to assess the inflammatory status of the eyelid margin^13^. Higher telangiectasia scores indicate greater extent of telangiectasia.

Non-invasive tear breakup time (TBUT) was then measured and meibography on the everted upper eyelid of the selected eye was captured using Oculus Keratograph®5 M (Wetzlar, Germany). Images of meibography were then exported to examine the meiboscore (glandular loss) using the Ngo 7 point scale^34^, and % of MG dropout measured using ImageJ software as previously described^35^. A lower TBUT indicates increased tear stability or greater tear evaporation.

A small amount of basal tear (0.5 μl) was collected using a disposable 0.5 μl glass microcapillary tube (Drummond Scientific, Broomall, PA) for tear lactoferrin concentration (mg/ml) using the commercially available point-of-care device TearScan^TM^ 270 MicroAssay System (Advance Tear Diagnostics, Birmingham, AL)^36^. Tear secretion was evaluated using a Schirmer strip without anesthesia, and read after 5 minutes. A lower Schirmer test result indicates relatively less tear secretion. Schirmer strips were then placed in a −80 °C freezer for batch analysis of tear eicosanoid concentrations (see lipid extraction, instrumentation and analysis sections).

One drop of topical anaesthetic (0.5% Alcain, Alcon, Japan) was then placed into the selected eye, and after eversion of the upper lid, an impression cytology sample was collected from the upper palpebral conjunctiva using a 12 mm Millicell tissue culture insert (model PICM01250, Merck Millipore, Ireland). After PAS staining, goblet cell density was counted and squamous metaplasia graded using the modified Nelson and Wright grading scheme^37^. Fluorescein dye was instilled and corneal staining graded with the modified Oxford grading scale^38^. An increased staining score indicates greater ocular surface damage, and greater severity of clinical dry eye disease.

### Tear eicosanoids extraction

The work flow of analysis is illustrated in Fig. S1. The wetted part of the Schirmer strip were cut into fine pieces (of approximately 2 mm) using micro scissors pre-washed with methanol and washed between samples. Lipids were released from the strips overnight at 4 °C with 1 mL of methanol (MeOH): water (H_2_O) (5:95 by v/v) at 900 rpm (revolution per minute) in a thermomixer (Eppendorf, Hamburg, Germany). To prevent oxidation, 20 μL of 0.1% butylated hydroxytoluene (BHT) was added. Samples were spiked with 50 μl of mixed deuterated internal standard solutions. Eicosanoid extraction was performed with some modifications of an earlier method^39^. The extraction recovery and linearity of representative standards are described in Fig. S2. Analytes were extracted using Strata-X 33 u polymer based solid reverse phase (SPE) extraction columns (8B-S100-UBJ, Phenomenex). Columns were conditioned with 3 mL of 100% MeOH and then equilibrated with 3 mL of H_2_O. After loading the sample, the columns were washed with H_2_O: MeOH: acetic acid (90:10:0.1 by v/v) to remove impurities, and the metabolites were then eluted with 2 times 500 µL of 100% MeOH. Prior to LC-MS/MS analysis, samples were evaporated using a SpeedVac and reconstituted in 50 µl of water-acetonitrile-acetic acid (60:40:0.02, v/v/v).

### Instrumentation

High-performance liquid chromatography (HPLC) was performed using an Agilent 1290 series chromatographer (Agilent, Santa Clara, USA). Reversed phase separation was performed on an Acquity UPLC BEH shield RP18 column (2.1 × 100 mm; 1.7 m; Waters) and maintained at 40 °C. The mobile phase consisted of (A) ACN/water/acetic acid (60/40/0.02, v/v) and (B) ACN/IPA (50/50, v/v). The stepwise gradient conditions were carried out for 10 min as follows: 0–5.0 min, 1–55% of solvent B; 5.0–5.5 min, 55–99% of solvent B, and finally 5.5–6.0 min, 99% of solvent B. The flow rate was 0.5 mL/min, injection volume was 10 µl, and all samples were kept at 4 °C throughout the analysis.

The HPLC system was coupled to Agilent 6495 triple-quad mass spectrometer (Agilent, Santa Clara, USA). The electrospray ionization was conducted in negative mode with the capillary and nozzle voltage set at 4000 V and 1000 V, respectively. Drying gas temperature was set at 250 °C with a gas flow of 12 L/min. Sheet gas temperature was set at 260 °C with a gas flow of 14 L/min. The nebulizer gas flow was 35 psi, high and low pressure RF at 150 and 60 V, respectively. The dynamic MRM option was used and performed for all compounds with optimized transitions and collision energies (Supp Table 1). The determination and integration of all peaks was manually performed using the MassHunter Workstation software (Agilent, Santa Clara, USA).

### Statistical analysis

The data were analyzed using SPSS version 24 (SPSS for Microsoft, Chicago, IL), and tested for normality using the Shapiro-Wilk test (p > 0.05). Differences in tear eicosanoids between the two age groups in the first experiment were examined using either independent T. For the second experiment, differences in tear eicosanoids between groups based on the meibomian gland expressibility score (case: <3 glands with liquid expressed, comparison: 3 or more glands with liquid meibum expressed) were examined using either independent T tests, as appropriate. Spearman tests (ρ) were carried out to assess the relationship between tear eicosanoids and clinical outcome measures. Diagnostic capability of tear eicosanoids to detect poor expressibility was determined using receiver-operating-characteristic curves (ROC) and the mean and 95% CI of area-under-curve (AUC) were calculated. All individual eicosanoids were evaluated separately using multiple linear regression models. Eicosanoids were selected for inclusion in the simultaneous multiple linear regression where correlations with lipid mediators were significant at p < 0.05 and correlation coefficient >0.35. The tear level of each eicosanoid was used as the dependent variable, whereas demographic and clinical variables were used as independent variables. The final models were determined using the method of backward elimination followed by forward entry, and chosen based on the maximal number of significant factors. Significance was determined at p < 0.05.

## Electronic supplementary material

Supplementary Information
