# Supplementary material for: Tear eicosanoids in healthy people and ocular surface disease
Source: Sci Rep. 2018 Jul 26;8:11296. doi: 10.1038/s41598-018-29568-3 (PMC6062525; doi:10.1038/s41598-018-29568-3)
Supplement: Supplementary file 1 — Supplementary Information [file 41598_2018_29568_MOESM1_ESM.docx]

**Tear eicosanoids in healthy people and ocular surface disease**

Yohannes Abere Ambaw^1,2^, Cecilia Chao^3,4,5^, Shanshan Ji^2^, Manfred Raida^2^, Federico Torta^1,2^, Markus R. Wenk^1,2,6^, Louis Tong ^3,7,8,9^

**Affiliations:**

^1^Department of Biochemistry, Yong Loo Lin School of Medicine, National University of Singapore

^2^Singapore Lipidomics Incubator, Life Sciences Institute, National University of Singapore, Singapore

^3^Ocular Surface Research Group, Singapore Eye Research Institute, Singapore

^4^The School of Optometry and Vision Science, University of New South Wales, Australia

^5.^Center For Translational Ocular Immunology, Department of Ophthalmology and Cornea Service, Tufts Medical Center, Boston, MA

^6^Department of Biological Sciences, National University of Singapore, Singapore

^7^Department of Ophthalmology, Yong Loo Lin School of Medicine, National University of Singapore, Singapore

^8^Department of Cornea and External Eye Disease, Singapore National Eye Center, Singapore

^9^Duke-NUS Medical School, Singapore

**Correspondence and reprints:**

Louis Tong MD PhD

Senior Consultant

Principal Clinician Scientist

Singapore National Eye Centre

The Academia, 20 College Road

Discovery Tower Level 6

Singapore 169856

Email: [louis.tong.h.t@singhealth.com.sg](mailto:louis.tong.h.t@singhealth.com.sg)

Tel: +65 98186221

Fax: +65 6225 2568

**Keywords:** Eicosanoids; Lipidomics, Dry eye; Meibomian gland dysfunction; Tear; Clinical study; Ocular surface inflammation.

**Supplementary materials**

**Table S1.** List of MRM transitions for eicosanoids species, and non-natural compounds used as internal standards (ISTD). An appropriate internal standard for the quantification each endogenous lipids are listed. The retention time, collision energy also reported for each compound. (M1 = precursor ion; M2 = product ion; RT=Retention time in minute and CE=collision energy).

| **Compound Name** | | **M1** | **M2** | **ISTD** | **RT** | **CE** | |
| --- | --- | --- | --- | --- | --- | --- | --- |
| (d4) 6k PGF1 | 373 | 167 | ISTD | 0.87 | 25 | |  |
| (d4) TXB_2_ | 373 | 173 | ISTD | 1.17 | 13 | |  |
| (d4) PGF_2α_ | 357 | 197 | ISTD | 1.26 | 25 | |  |
| (d4) PGE_2_ | 355 | 275 | ISTD | 1.31 | 13 | |  |
| (d4) PGD_2_ | 355 | 193 | ISTD | 1.47 | 13 | |  |
| (d4) dhk PGF_2α_ | 357 | 187 | ISTD | 1.68 | 21 | |  |
| (d4) dhk PGD_2_ | 355 | 179 | ISTD | 1.94 | 19 | |  |
| (d4) 5-iso PGF_2α_ VI | 364 | 115 | ISTD | 1.14 | 21 | |  |
| (d4) LTB_4_ | 339 | 197 | ISTD | 2.79 | 13 | |  |
| (d8) 5-HETE | 327 | 116 | ISTD | 4.65 | 13 | |  |
| (d8) 12-HETE | 327 | 184 | ISTD | 4.47 | 13 | |  |
| (d8) 15-HETE | 327 | 226 | ISTD | 4.22 | 13 | |  |
| (d4) 9-HODE | 299 | 172 | ISTD | 4.29 | 19 | |  |
| (d4) 13-HODE | 299 | 198 | ISTD | 4.28 | 13 | |  |
| (d4) Resolvin E1 | 353 | 197 | ISTD | 0.96 | 13 | |  |
| (d11) 8,9-EET | 330 | 155 | ISTD | 4.92 | 9 | |  |
| (d11) 11,12-DHET | 348 | 167 | ISTD | 3.4 | 19 | |  |
| (d11) 14,15-EET | 330 | 175 | ISTD | 4.78 | 9 | |  |
| (d4) 9,10-diHOME | 317 | 203 | ISTD | 3.15 | 21 | |  |
| (d4) 12,13-diHOME | 317 | 185 | ISTD | 3.05 | 21 | |  |
| (d5) LTE4 | 443 | 338 | ISTD | 2.1 | 17 | |  |
| (d8) Arachidonic acid | 311 | 267 | ISTD | 5.97 | 13 | |  |
| TxB_2_ | 369 | 169 | (d4) TXB_2_ | 1.12 | 13 | |  |
| PGF_2α_ | 353.3 | 193 | (d4) PGF_2α_ | 1.2 | 25 | |  |
| PGE_2_ | 351 | 271 | (d4) PGE_2_ | 1.31 | 13 | |  |
| PGD_2_ | 351 | 271 | (d4) PGD_2_ | 1.49 | 13 | |  |
| tetranor 12-HETE | 265 | 109 | (d4) LTB_4_ | 3.11 | 9 | |  |
| 12-HHTrE | 279 | 217 | (d4) LTB_4_ | 3.33 | 13 | |  |
| 11-HETE | 319 | 167 | (d8) 5-HETE | 4.43 | 13 | |  |
| 11-HEPE | 317 | 167 | (d8) 5-HETE | 3.79 | 13 | |  |
| 13-HDoHE | 343 | 221 | (d8) 15-HETE | 4.41 | 13 | |  |
| 9-HETE | 319 | 123 | (d8) 5-HETE | 4.54 | 13 | |  |
| 9-HEPE | 317 | 149 | (d8) 5-HETE | 4.1 | 13 | |  |
| 8-HDoHE | 343 | 109 | (d8) 5-HETE | 4.55 | 13 | |  |
| 16-HDoHE | 343 | 233 | (d8) 15-HETE | 4.34 | 13 | |  |
| 20-HDoHE | 343 | 241 | (d8) 15-HETE | 4.24 | 9 | |  |
| LTB_4_ | 335 | 195 | (d4) LTB_4_ | 2.79 | 13 | |  |
| 6-trans-LTB_4_ | 335 | 195 | (d4) LTB_4_ | 2.52 | 13 | |  |
| 5,6-diHETE | 335 | 115 | (d4) LTB_4_ | 3.84 | 21 | |  |
| 5-HETE | 319 | 115 | (d8) 5-HETE | 4.66 | 13 | |  |
| 5-HEPE | 317 | 115 | (d8) 5-HETE | 4.05 | 13 | |  |
| 7-HDoHE | 343 | 141 | (d8) 5-HETE | 4.54 | 13 | |  |
| 4-HDoHE | 343 | 101 | (d8) 5-HETE | 4.81 | 9 | |  |
| 9-HOTrE | 293 | 171 | (d8) 5-HETE | 3.66 | 13 | |  |
| 6S-LXA_4_ | 351.3 | 115 | (d4) LTB_4_ | 1.89 | 9 | |  |
| Resolvin E1 | 349 | 195 | (d4) Resolvin E1 | 0.89 | 13 | |  |
| Resolvin D1 | 375 | 141 | (d4) Resolvin E1 | 1.74 | 13 | |  |
| Protectin D1 | 359 | 153 | (d4) Resolvin E1 | 1.85 | 13 | |  |
| 8,15-diHETE | 335 | 235 | (d4) LTB_4_ | 2.51 | 19 | |  |
| 15-HETE | 319 | 175 | (d8) 15-HETE | 4.34 | 13 | |  |
| 15-HEPE | 317 | 219 | (d8) 5-HETE | 3.81 | 13 | |  |
| 17 HDoHE | 343 | 229 | (d8) 15-HETE | 4.32 | 9 | |  |
| 13-HODE | 295 | 195 | (d4) 13-HODE | 4.27 | 13 | |  |
| 13-HOTrE | 293 | 195 | (d4) 13-HODE | 3.85 | 19 | |  |
| 15-HETrE | 321 | 221 | (d8) 15-HETE | 4.69 | 13 | |  |
| 8-HETE | 319 | 155 | (d8) 5-HETE | 4.54 | 9 | |  |
| 8-HEPE | 317 | 155 | (d8) 5-HETE | 4.04 | 21 | |  |
| 10-HDoHE | 343 | 153 | (d8) 5-HETE | 4.45 | 9 | |  |
| 8-HETrE | 321 | 157 | (d8) 5-HETE | 4.72 | 13 | |  |
| 12-HETE | 319 | 135 | (d8) 12-HETE | 4.43 | 13 | |  |
| 12-HEPE | 317 | 179 | (d8) 12-HETE | 3.88 | 9 | |  |
| 14-HDoHE | 343 | 205 | (d8) 15-HETE | 4.43 | 9 | |  |
| 11-HDoHE | 343 | 149 | (d8) 15-HETE | 4.58 | 11 | |  |
| 9-HODE | 295 | 171 | (d4) 9-HODE | 4.26 | 19 | |  |
| 12-oxoETE | 317 | 153 | (d8) 15-HETE | 4.42 | 13 | |  |
| 15-oxoETE | 317 | 113 | (d8) 15-HETE | 4.33 | 13 | |  |
| 20-HETE | 319.3 | 275 | (d8) 15-HETE | 3.84 | 13 | |  |
| 19-HETE | 319 | 231 | (d8) 15-HETE | 3.78 | 13 | |  |
| 18-HETE | 319 | 261 | (d8) 15-HETE | 3.82 | 9 | |  |
| 17-HETE | 319 | 247 | (d8) 15-HETE | 4.11 | 9 | |  |
| 16-HETE | 319 | 189 | (d8) 15-HETE | 4.03 | 9 | |  |
| 18-HEPE | 317 | 215 | (d8) 15-HETE | 3.7 | 9 | |  |
| 5,6-EET | 319 | 191 | (d11) 8,9-EET | 5.19 | 9 | |  |
| 8,9-EET | 319 | 123 | (d11) 8,9-EET | 5.07 | 9 | |  |
| 11,12-EET | 319 | 167 | (d11) 14,15-EET | 4.95 | 9 | |  |
| 14,15-EET | 319 | 219 | (d11) 14,15-EET | 4.78 | 9 | |  |
| 19(20)-EpDPE | 343 | 241 | (d8) 15-HETE | 4.7 | 9 | |  |
| 19,20-DiHDPA | 361 | 229 | (d11) 11,12-DHET | 3.28 | 13 | |  |
| 5,6-diHETrE | 337.3 | 145 | (d11) 11,12-DHET | 3.82 | 13 | |  |
| 8,9-diHETrE | 337.2 | 127 | (d11) 11,12-DHET | 3.65 | 19 | |  |
| 11,12-diHETrE | 337.3 | 167 | (d11) 11,12-DHET | 3.53 | 13 | |  |
| 14,15-diHETrE | 337.3 | 207 | (d11) 11,12-DHET | 3.24 | 13 | |  |
| 9,10-diHOME | 313 | 201 | (d4) 9,10-diHOME | 3.19 | 21 | |  |
| 12,13-diHOME  9,10-EpOME  12,13-EpOME | 313  295  295 | 183  171  195 | (d4) 12,13-diHOME  (d11) 8,9-EET  (d11) 8,9-EET | 3  4.8  4.75 | 21  13  13 | |  |
| Arachidonic acid | 303 | 259 | (d8) Arachidonic acid | 5.92 | 13 | |  |
| Adrenic acid | 331 | 287 | (d8) Arachidonic acid | 6.03 | 13 | |  |
| EPA | 301 | 257 | (d8) Arachidonic acid | 5.78 | 9 | |  |
| DHA | 327 | 283 | (d8) Arachidonic acid | 5.93 | 9 | |  |
|  | |  |  |  |  |  |  |


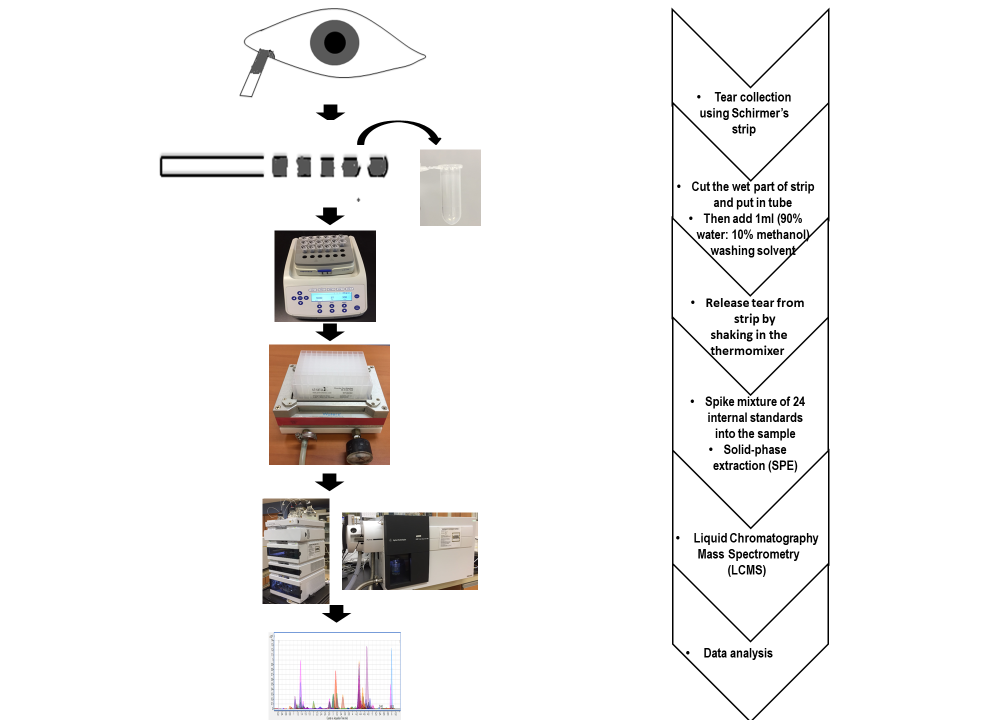


**Figure S1**. Workflow for eicosanoids analysis. Tears were collected from subjects using the Schirmer’s strip for 5 min and then wetted part of the strip were cut into fine pieces. Lipids were released from the fine pieces strips overnight shaking in a thermomixer with washing solvent. Samples were spiked with 50 μl of mixed deuterated internal standard solutions (0.03 µg/mL for 23 IS and 2 ug/mL for d8-AA). Eicosanoids were extracted by SPE with pure methanol as elution solvent. Total extracts reconstituted and inject into a LCMS. SPE: solid phase extraction, IS: internal standard, d8-AA: deuterated arachidonic acid, LCMS: liquid chromatography mass spectrometry.


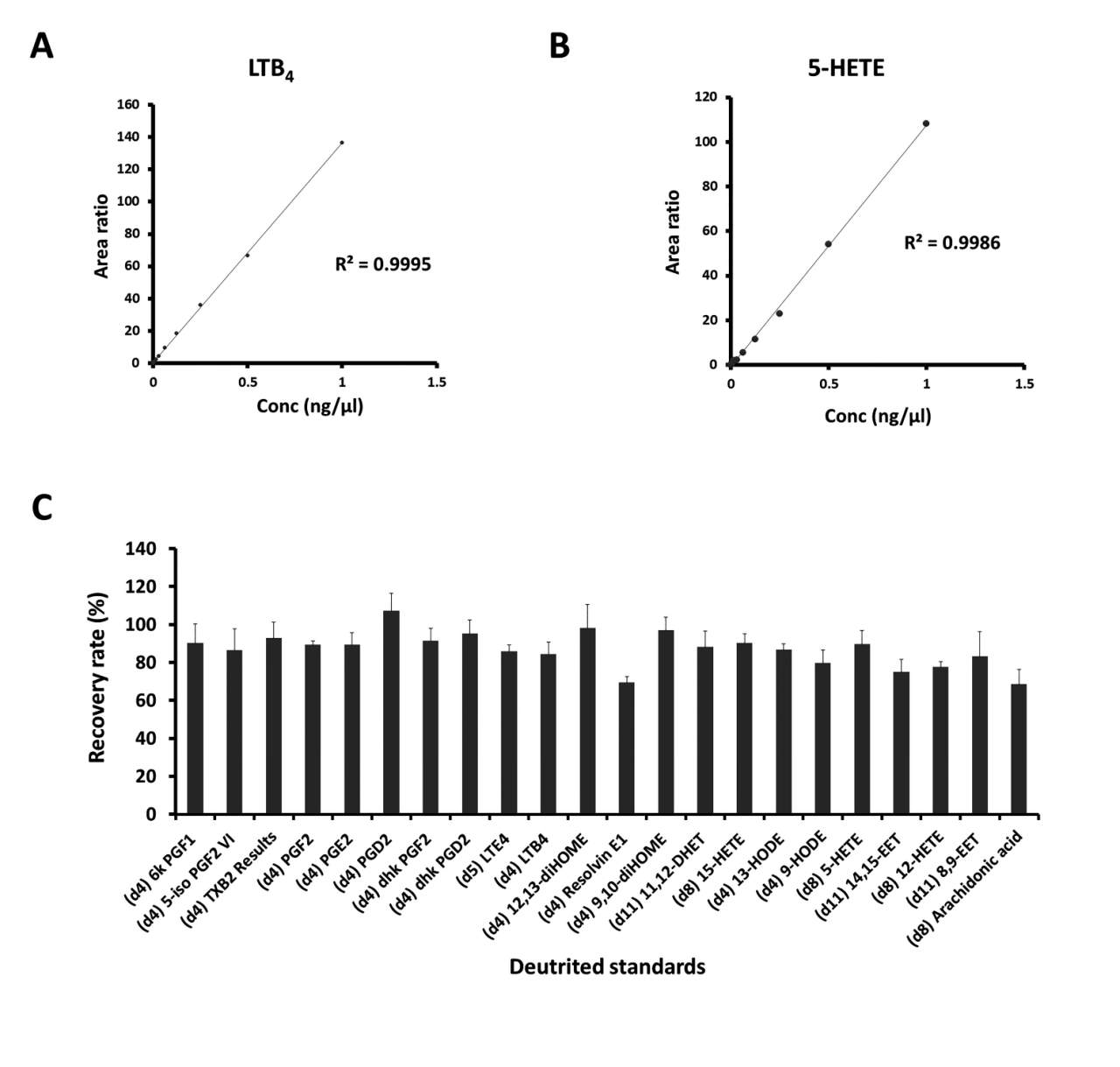


**Figure S2**. Linearity and recovery rate. A and B, line graphs, shows linearity of representative standards LXB_4_ and 5-HETE respectively. Y axis indicate ratio of analyte peak area to internal standard peak area and X axis indicate the concentration of the analyte standard. C. Extraction recovery rate of deuterated standards. Mean values were plotted. Error bars indicate standard errors of the means.


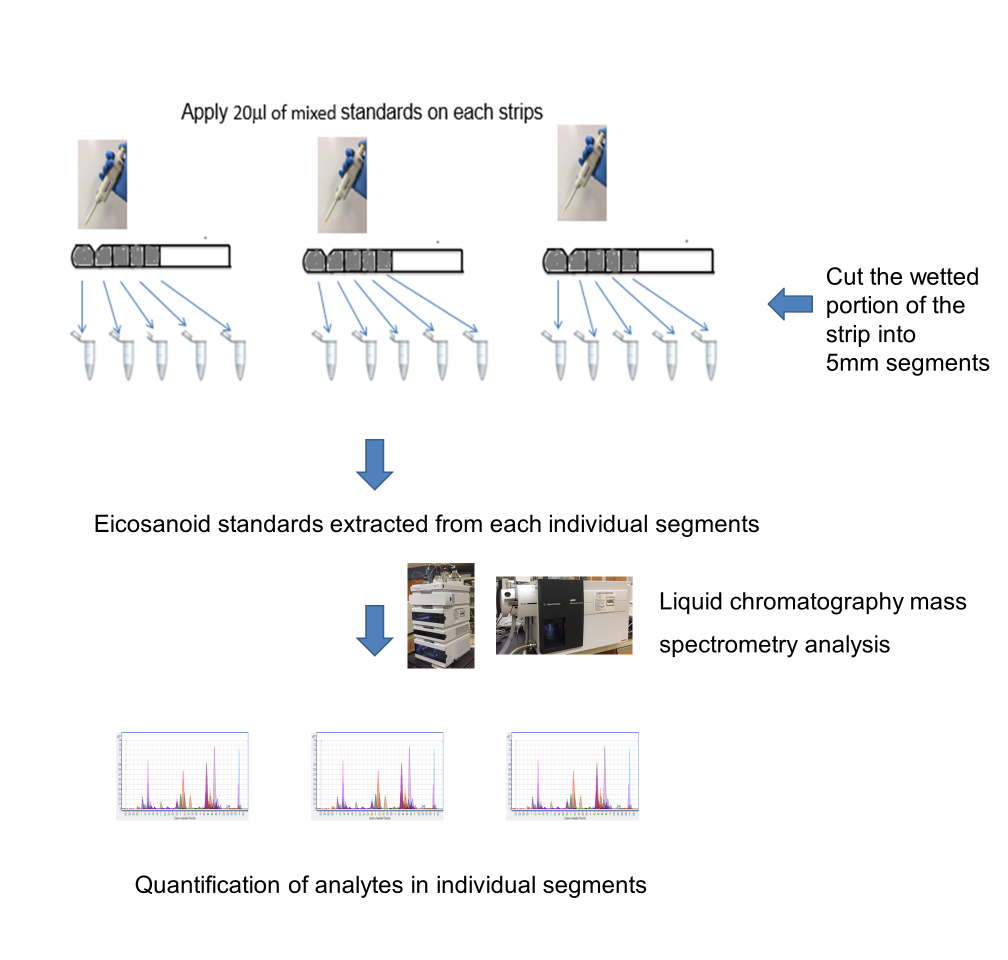


**Figure S3.** Schematic diagram illustrating the procedures for validating the gradient of metabolites along the wetted length of the strips. Briefly, mixed non-deuterated standards were applied on the edge of the Schirmer’s strip and the wetted portions of the strips were cut into 5 mm. Lipids were then extracted from individual segments and analyzed.


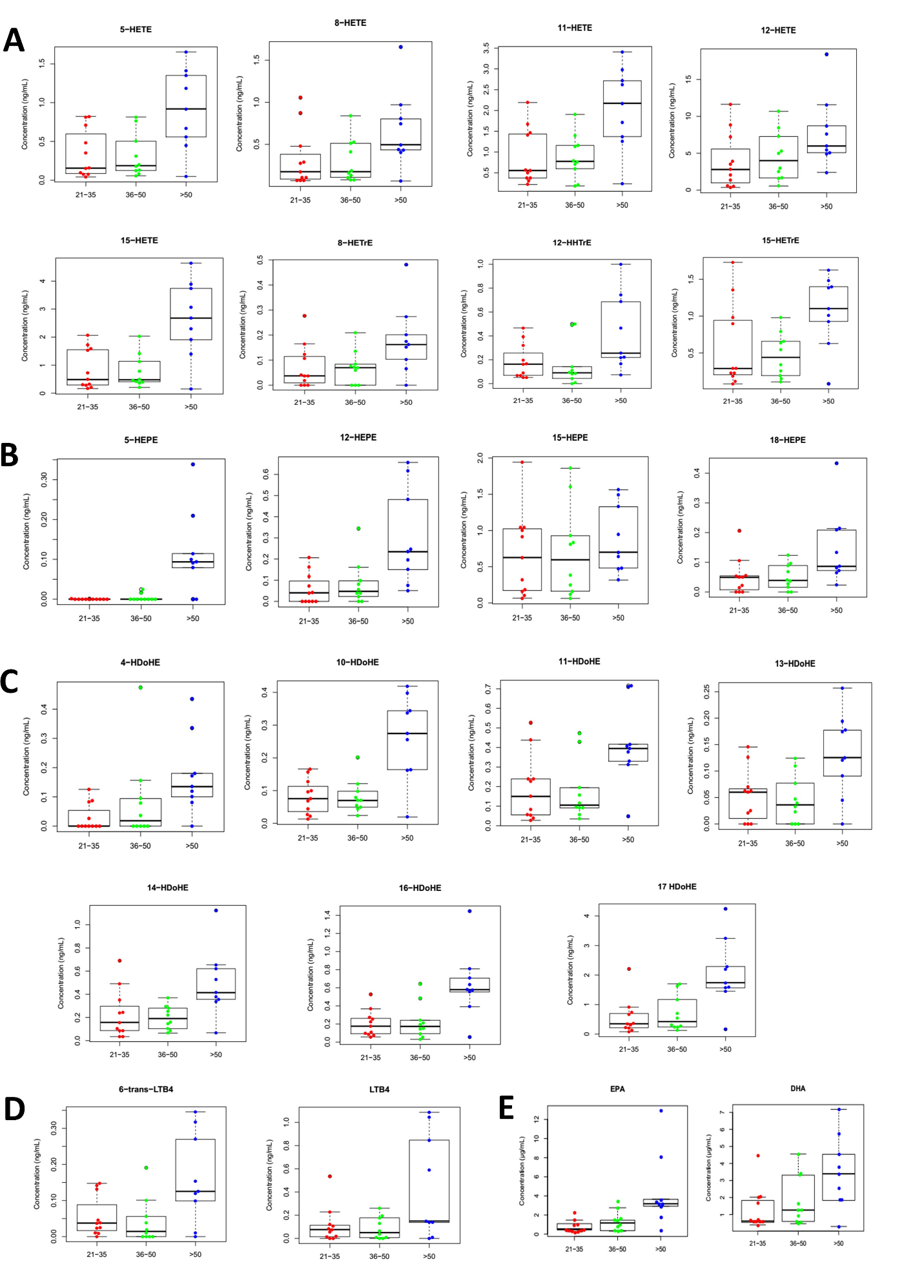


**Figure S4**. Box plots showing the median (bold horizontal line), interquartile rang (box) and eicosanoids concertation levels in the Y-axis across three different healthy individuals age groups (21-35, 35-50, >50). **A**. HETEs (5,8,11,12,15) and HETrE (8,12,15), **B**. HEPEs (5,12,15,18), **C.** HDoHEs (4,10,11,13,14,16,17), **D.** LTBs, and

**E**. Omega-3 fatty acids.


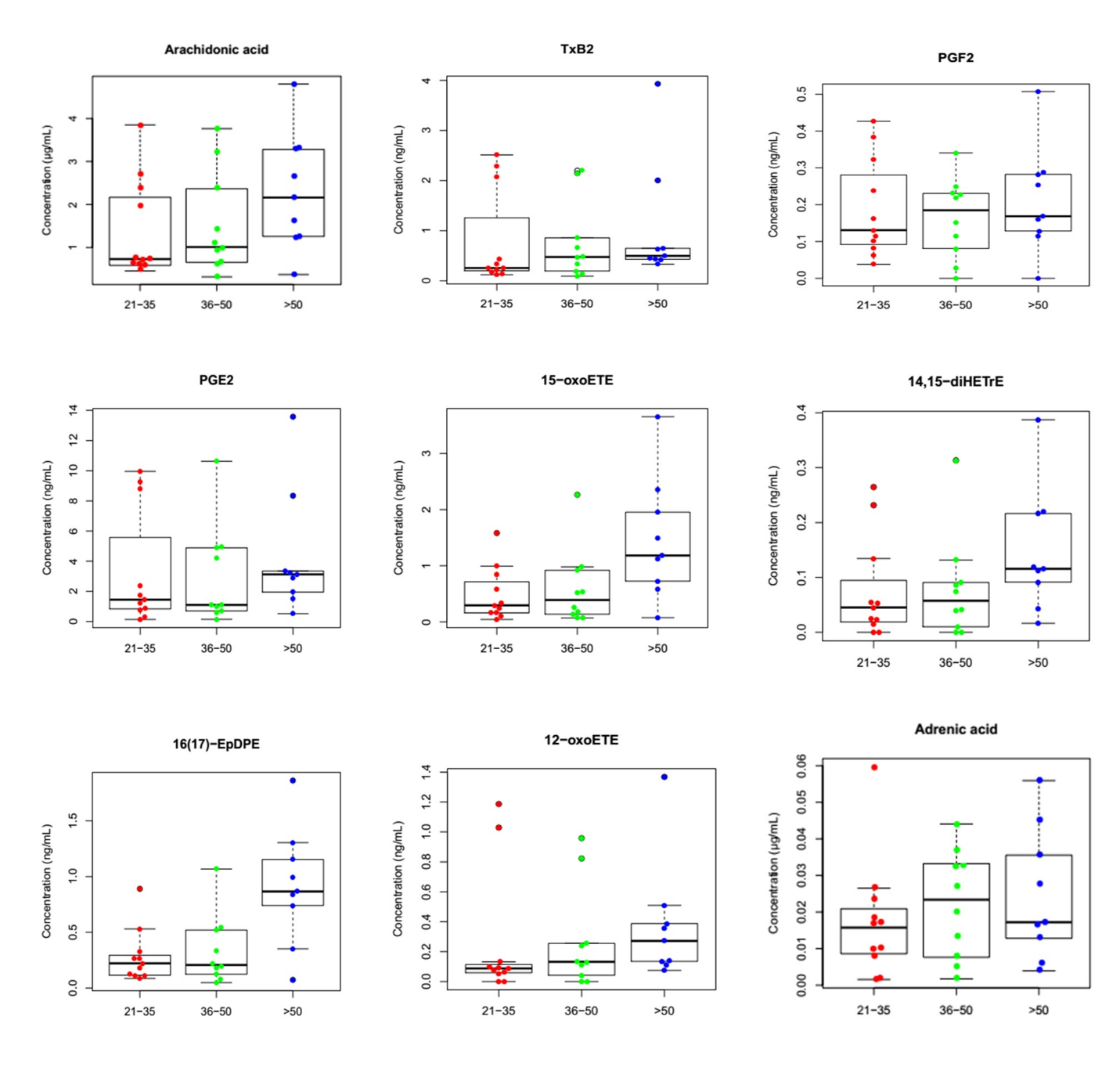


**Figure S5:** Box plots showing the median (bold horizontal line), interquartile rang (box) and eicosanoids concertation levels in the Y-axis across three different healthy individuals age groups (21-35, 35-50, >50). There is no significance difference between the groups in 10 compounds.


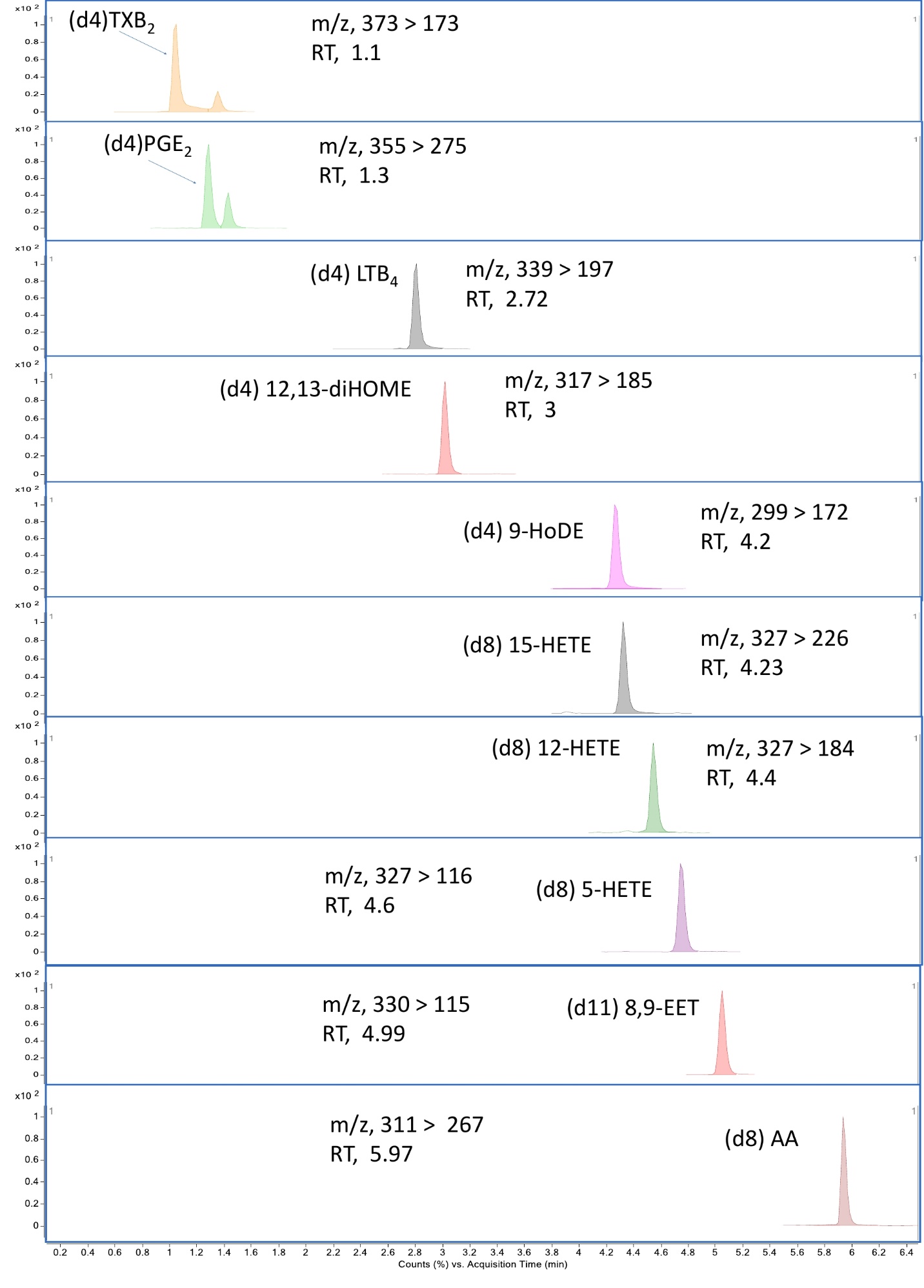


A.


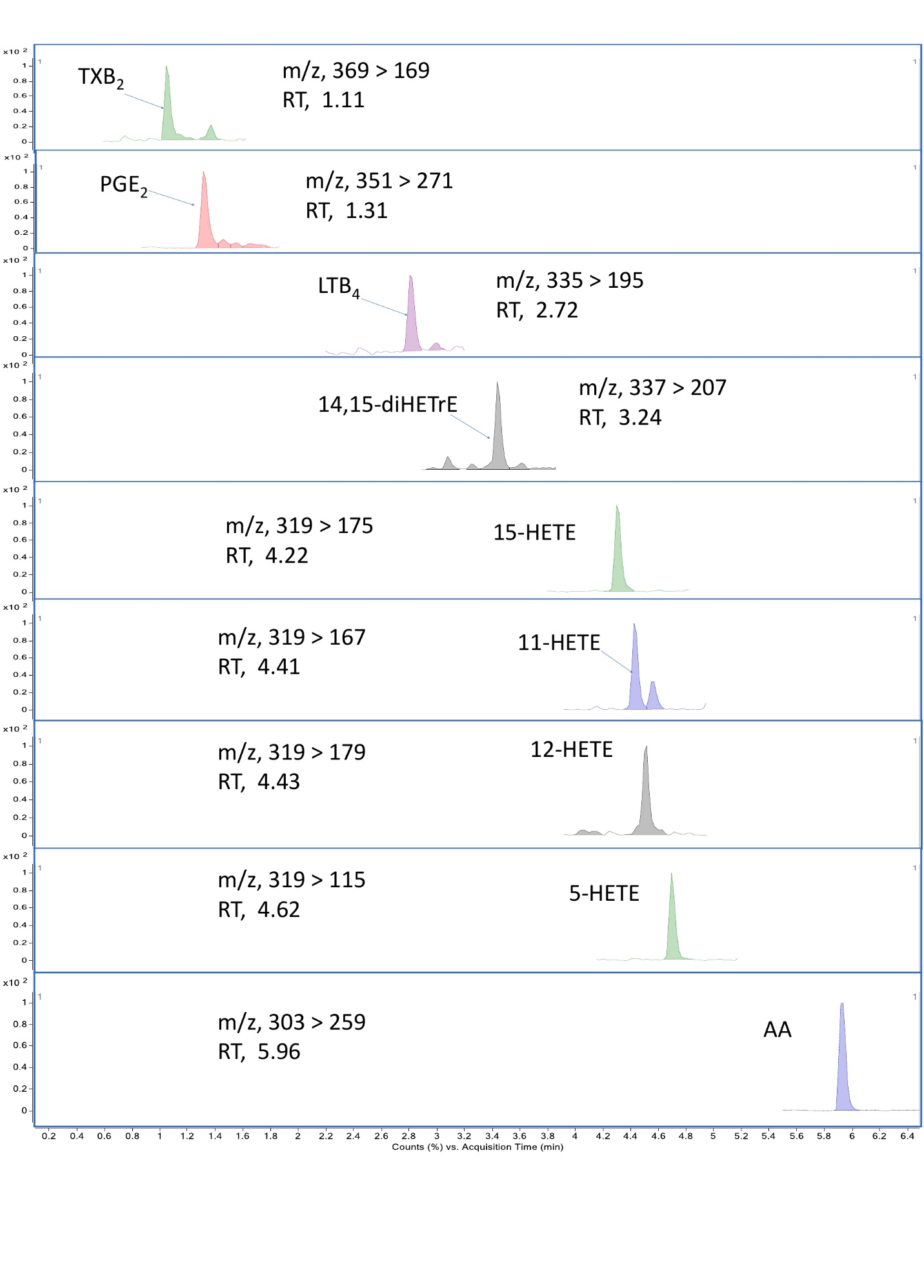


B.

**
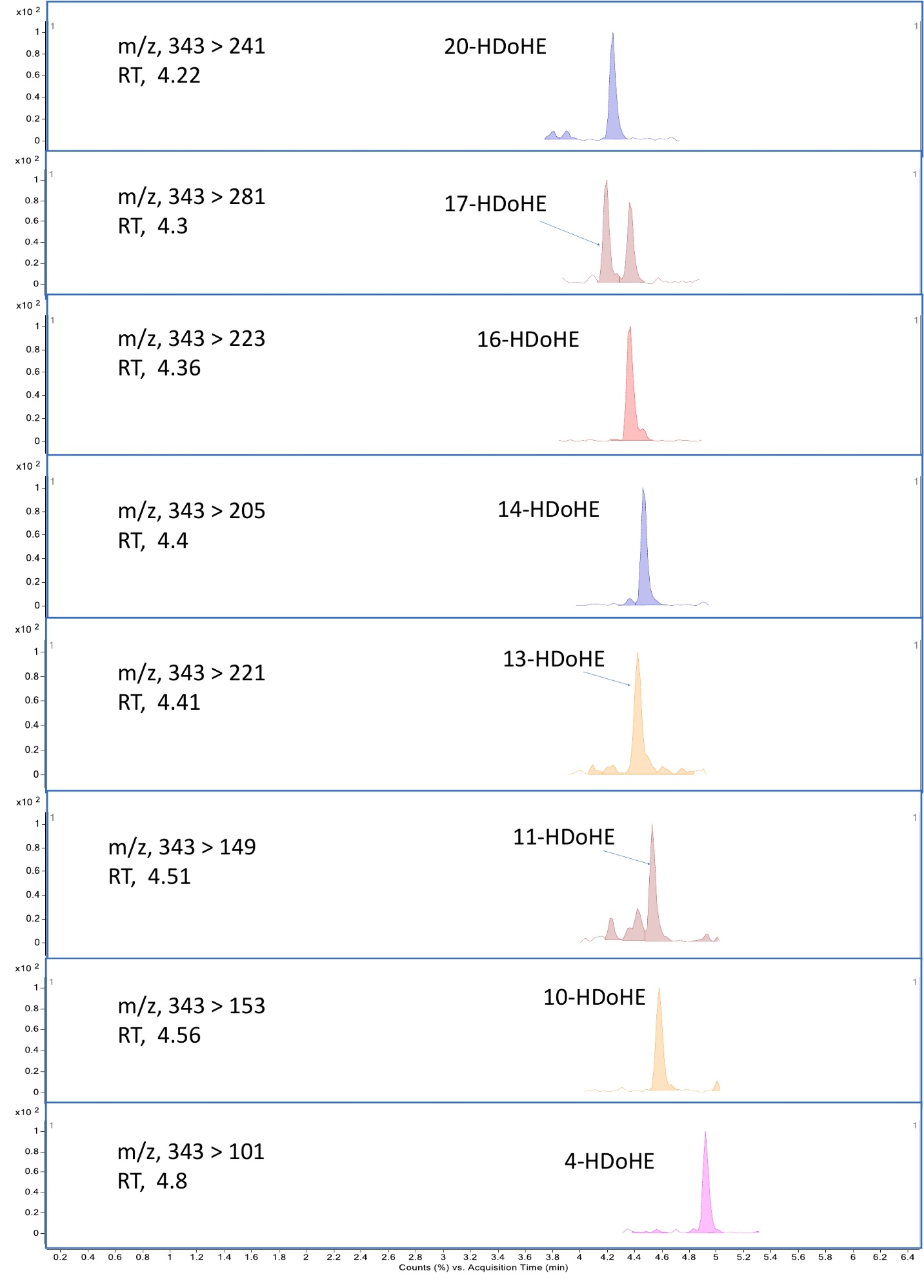
**

C.

**Figure S6.** Chromatographic profiles of selected endogenous eicosanoids in human tears and synthetic standards. The signals were obtained by LC-MSMS (MRM mode). A) Chromatograms of selected internal standards. (B) Chromatograms of prostaglandin E_2_ (PGE_2_), Thromboxane B_2_ (TXB_2_), Leukotriene B_4_ (LTB_4_), hydroxyeicosanoids (HETEs) and Arachidonic acid (AA). (C) Representative chromatograms of the hydroxydocosahexaenoic acids.


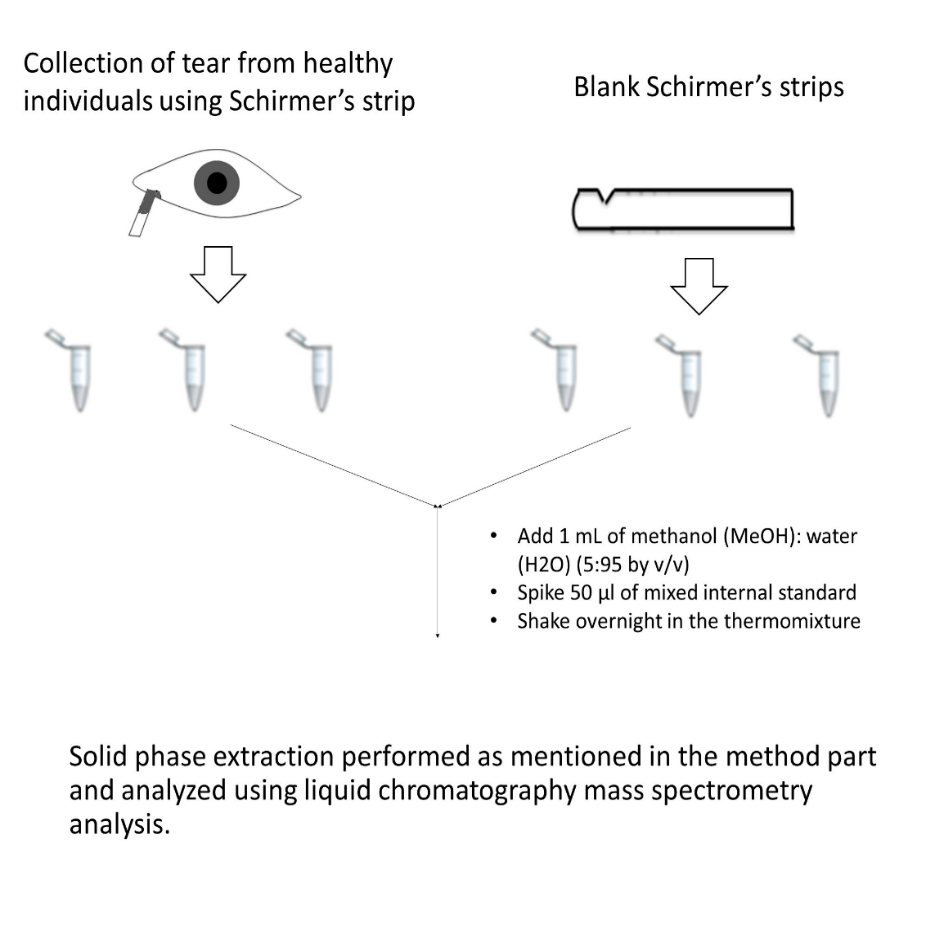


**Figure S7.** Schematic diagram illustrating the extraction of eicosanoids from tear containing Schirmer’s strip and blank Schirmer’s strips.


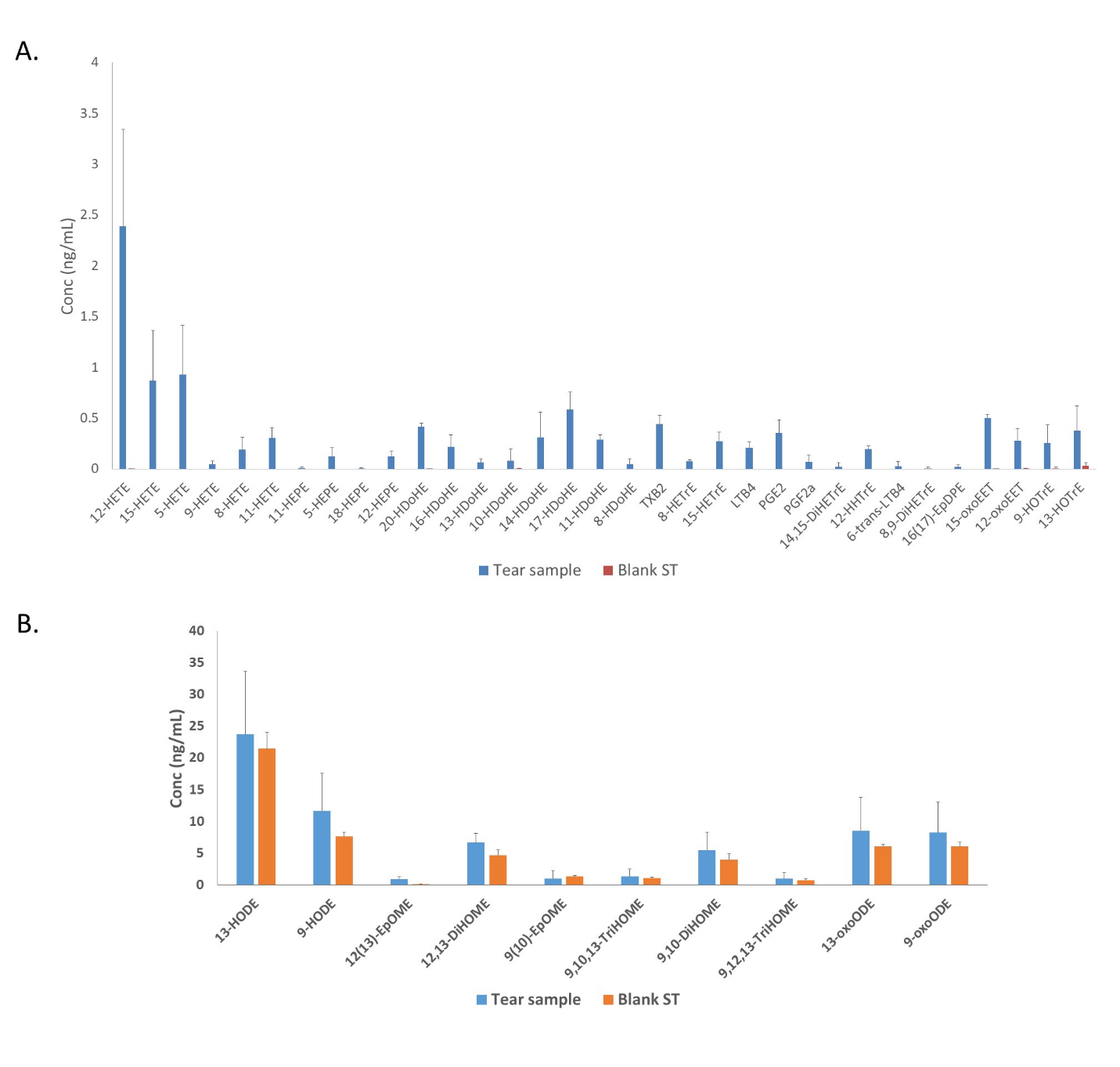


**Figure S8.** Bar chart shows the background effect of the paper in the analysis of eicosanoids. (A) Compounds not influenced by blank Schirmer’s strips (B) compounds influenced by blank Schirmer’s strip. Mean values were plotted. Error bars indicate standard errors of the means. ST: Schirmer’s tears.


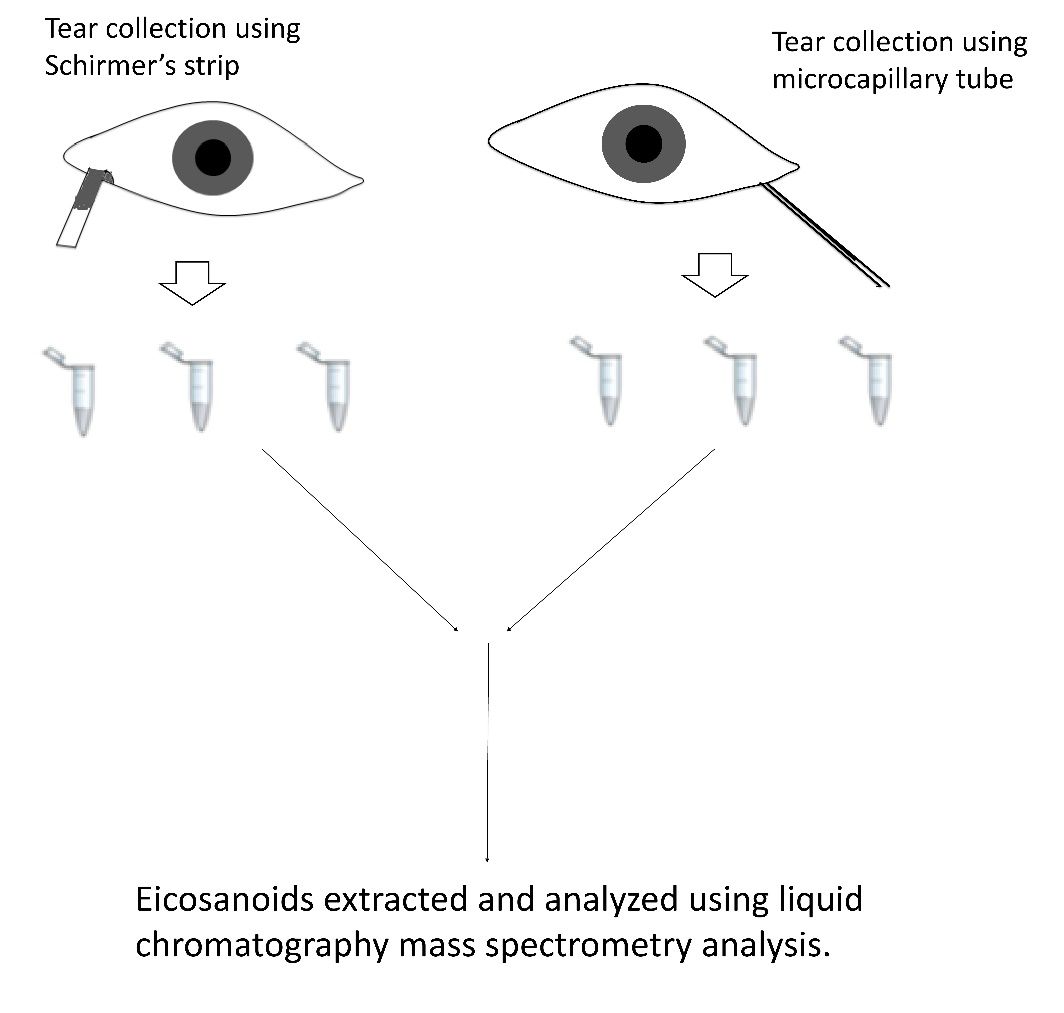


**Figure S9.** Schematic diagram illustrating the procedures for Schirmer’s strips and Microcapillary tear collection used for this comparison. Capillary tears were collected using 0.5 µl glass microcapillary tubes applied gently to the temporal margin of the lower eyelid. A minimum of 10 to 15 µL tears was collected from 3 healthy individuals and released by bulb dispenser into tubes. Schirmer’s strip tears were collected from three different subjects.


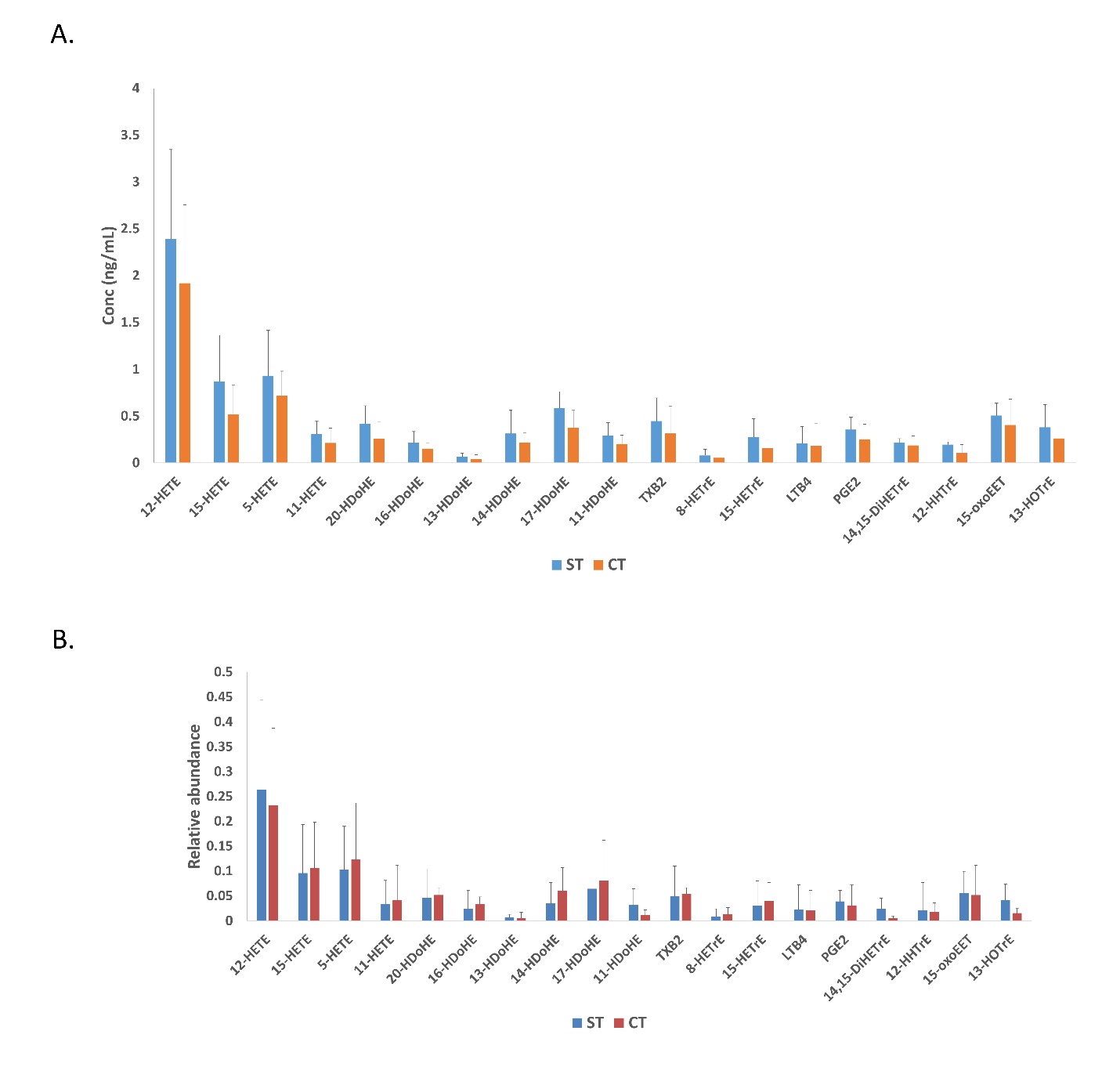


**Figure S10.** Bar charts showing the comparison of eicosanoids levels obtained with two different tear collection techniques, Schirmer’s strip and capillary tube.  (A) Absolute concentration of eicosanoids in tear samples collected using the Schirmer’s strips (n= 3) and capillary tubes (n=3). (B) The relative abundance of eicosanoids found in tear samples collected using Schirmer’s strips and capillary tubes. Mean values were plotted. Error bars indicate standard deviation of the means. ST: Schirmer’s tears; CT: capillary tears.
